# Characterizing pre-discharge interventions to reduce length of stay for older adults: A scoping review

**DOI:** 10.1371/journal.pone.0318233

**Published:** 2025-02-10

**Authors:** Emily Garcia, Zachary J. Hass

**Affiliations:** 1 School of Industrial Engineering, Purdue University, West Lafayette, IN, United States of America; 2 Regenstrief Center for Healthcare Engineering, Purdue University, West Lafayette, IN, United States of America; 3 School of Nursing, Purdue University, West Lafayette, IN, United States of America; University of California San Diego, UNITED STATES OF AMERICA

## Abstract

**Background:**

Hospital pre-discharge interventions are becoming one of the leading strategies to promote early discharge. For older adult patients, it remains unclear what these interventions are and how they affect discharge outcomes.

**Objective:**

This scoping review categorizes pre-discharge interventions promoting early acute care hospital discharging or total hospital length of stay reductions among older adults, synthesizes contextual factors (e.g., cost, staffing) driving implementation, and assesses the perceived intervention’s impact.

**Design:**

The review followed the five states of the Arksey and O’Malley framework and the PRISMA-ScR extension. The PubMed, Embase, and Scopus databases were searched from 1983 to 2020 for pre-discharge interventions designed or adapted to discharge older adults earlier in their stay from acute care hospitals. Potentially relevant articles were screened against eligibility criteria. Findings were extracted and collated in data charting forms followed by brief thematic analyses.

**Results:**

The search yielded 5,455 articles of which 91 articles were included. Eight pre-discharge intervention categories were identified: clinical management, diagnostic/risk assessment tools, staffing enhancements, drug administration, length of stay protocols, nutrition planning, and communication improvements. Leading motivations for intervention implementation included the nationwide drive to reduce care costs and hospitals’ need to increase hospital profitability, improve quality of care, or optimize resource utilization. Discharge outcomes reported included hospitalization costs, readmission rates, mortality rates, resource utilization rates and costs, and length of stay. Mixed results were found regarding the effectiveness of early discharge interventions on discharge outcomes based on expressed author sentiment.

**Conclusions:**

The drive for pre-discharge interventions that reduce older adult hospital stays and associated costs continues to stem primarily from economic and governmental policies. Follow-up studies may be required to emphasize patient perspectives and care trajectories to avoid unintentional costly and health-deteriorating consequences.

## Introduction

The 1983 establishment of the Prospective Payment System (PPS) by the Centers for Medicare and Medicaid Services (CMS) spurred a nationwide effort to promote cost-efficient hospital management to curb rapidly increasing care costs [1–7]. Hospitals became motivated to discharge patients sooner, nearing the lower length of stay (LOS) range permitted by PPS, to reduce resource utilization and maximize revenue [8–14]. PPS offered hospitals a fixed price for diagnosis-related care, enabling hospitals to generate net revenue from hospitalizations with lower care costs than the PPS reimbursed for [15].

Within the first three years of the PPS, average national hospital LOS decreased by 25%. For older adults undergoing surgical procedures the mean LOS decrease from 21.9 days to 12.6 days [9, 11, 16]. Hospitals also increased patient referrals to post-acute care services from 38% to 60%, shifting recovery care locations to nursing homes and other settings. This improved bed turn-over rates, optimized hospital resource usage, and secured shorter patient stay rewards from some insurance payers, enabling another source of hospital revenue [8, 13, 14, 16, 17].

However, the financial incentive to decrease unnecessary care services may cause hospitals to choose the least expensive or minimal number of care procedures, raising concerns over reduced patient care quality. Retrospective studies identified that older adult inpatients have suffered a significantly higher rate of poor care quality correlating to LOS reductions, suggesting that curbing healthcare costs has had unintended negative consequences [18]. Researchers have also identified increases in negative post-discharge outcomes such as hospital readmissions, mortality, and health deterioration [11, 12, 16, 17, 19–21]. Kosecoff et al. (1990) found that the proportion of patients discharged home in unstable conditions increased from 10% before the PPS (1981–1982) to 15% shortly afterward (1985–1986) [5]. This gave rise to the term “quicker and sicker” referring to the trend of hospital patients discharging sooner than the average LOS for their respective diagnosis while less stable than was previously customary [22–26]. From a patient perspective, reductions in LOS have generated feelings of pressure to leave the hospital with suboptimal care, diagnostic errors, poor communication and information exchange [1, 2, 8].

Currently, early discharges are attributed to additional causes besides economic and policy pressures. Enhanced recovery programs, early mobility programs, and discharge planning strategies have been developed to reduce hospital LOS. Literature reviews on hospital interventions driving early discharges or reduced LOS and their impact on patient outcomes and hospitalization costs remains limited. Coffey et al. (2019) and Siddique et al. (2020) are presently the only two systematic reviews on early discharge interventions [27, 28]. Neither systematic review focuses on pre-discharge interventions (interventions occurring before the discharge planning stage) associated with acute care hospital early discharging. Additionally, no single review on pre-discharge interventions targets explicitly older adults, a high-risk population for hospital readmissions, mortality, and health deterioration due to hospitalization.

In this paper, we review acute care pre-discharge interventions promoting or indirectly influencing early discharging of older adult patients. We also synthesize contextual factors driving their development and implementation and assess author sentiment on the perceived intervention’s impact.

### Research questions

The research questions guiding this scoping review were:

1. What type of pre-discharge interventions were developed and implemented to promote or indirectly drive early discharge or decreases in LOS of older adult patients in hospitals?
2. What motivates the development and implementation of these interventions?
3. What is the author’s sentiment regarding the impact of these interventions?

## Materials and methods

This scoping review was guided by the Arksey and O’Malley five-stage framework [29]. This methodology summarizes the available evidence on a topic, mapping the existing literature in a field of interest to convey its breadth and depth, including the volume, nature, and characteristics of primary research, while also identifying gaps. Following the methodology of scoping reviews, we did not formally evaluate the studies’ quality.

We disclose that this scoping review’s protocol was not pre-registered due to the inherent methodological flexibility inherent to this research design. Scoping reviews are exploratory in nature, often requiring iterative refinement of the research question and search strategy, which makes advance registration challenging. Nonetheless, to ensure rigor and transparency, we adhered to established methodological frameworks, following best practices recommended by Arksey and O’Malley, as previously mentioned. This approach maintained scientific integrity throughout the review process.

The review included the following key stages: (1) identifying the research question; (2) identifying relevant studies; (3) selecting relevant search studies; (4) charting the data and (5) collating, summarizing, and reporting results. We also applied the checklist for the Preferred Reporting Items for Systematic Reviews and Meta-Analyses Extension for Scoping Reviews (PRISMA-ScR) to ensure a comprehensive and systematic approach (see S1 Checklist in Supporting Information) [30]. However, the review protocol was not registered in a publicly accessible database prior to the commencement of the review.

### Search strategy

Articles in PubMed, Scopus, and Embase, published from 1983 to September 2020, were retrieved using the following keywords: early discharge, reduced length of stay, pre-discharge intervention, acute care, hospitalization, and older adults. The age criteria for ‘older adults’ were defined by the articles themselves. Exclusion keywords that were added to refine the search strategy in PubMed included: telephone, telemedicine, labor, mother, early follow-up, mental health, psychiatric, delayed discharge, and follow-up. The start date of 1983 was selected to identify pre-discharge hospital interventions developed and implemented after the PPS establishment. The end date was selected to create a buffer period that would ensure studies were not influenced by the COVID-19 pandemic, which had a significant impact on hospital management and care delivery [31]. To further support this approach, the specific search strategies for each database are provided in S1 Appendix.

### Selection criteria

Inclusion and exclusion criteria were refined after the initial article pull. Articles were eligible if they reported on early discharge, premature discharge, or reduced LOS associated with any type of intervention developed and implemented before the discharge planning phase among older adults in U.S. acute hospitals and were published in English. Our interest was in studying the U.S. healthcare system. There was no additional restriction on study design, hospital diagnosis, or patient characteristics. Exclusion criteria included articles focusing on surgical interventions emphasizing procedural innovations rather than hospital care management improvement efforts. Interventions related to hospital transfers were excluded, as they do not represent overall early discharge from acute care. Additionally, interventions for patients with psychiatric conditions were excluded, as physical and mental health acute care stays are fundamentally distinct. Articles concerning follow-up care, hospice care, advance care planning, discharging against medical advice, or telehealth/telemedicine were also excluded.

### Selection process

Studies were selected through a two-step process. First, abstracts were collated in Rayyan, a collaborative free web and mobile app tool, where both reviewers independently screened titles and abstracts [32]. Then, potentially eligible articles were assessed at the full-text level by both reviewers. Studies without accessible full-texts, those behind paywalls, or those not discussing hospital-driven interventions were excluded, with reasons for exclusion noted. Any discrepancies between reviewers were resolved through discussion.

### Data charting

We developed a data dictionary detailing information to collect, for consistency between reviewers throughout charting. Each reviewer extracted key data elements, including authors, publication year, study design, intervention description and motivation, targeted population, cohort size (if applicable), measured study outcomes, and authors’ sentiment and reasoning regarding early discharge. To enhance understanding of the study population and the intervention’s effect size, a single reviewer extracted additional data elements, including gender distribution (if available), average age (if available), and quantitative outcome results, as this task was deemed manageable for a single reviewer. One of the two reviewers checked the data extracted for consistency throughout the charting process. The quality of the extracted data was not appraised, as this was out of the scope of our review’s objectives.

### Author sentiment

Early discharge sentiment was defined on a scale of positive, negative, and neutral. Positive early discharge sentiment indicated cost reductions, improvements in patient outcomes, or similar measurements being highlighted by the author. Negative sentiment derived from increased costs, increased patient adverse outcomes, or similar measurements being mentioned. Neutral sentiment reflected a mix of positive and negative outcomes outlined by the author.

### Data analysis and synthesis

We conducted a thematic analysis of extracted data frequencies to identify predominant categories of pre-discharge interventions. Next, we conducted a content analysis using extracted data to synthesize contextual factors motivating the development and implementation of pre-discharge interventions for early discharge of older adults. Author sentiment was also assessed via content analysis. Finally, reviewers met as needed to discuss key findings.

## Results

**Fig 1:**
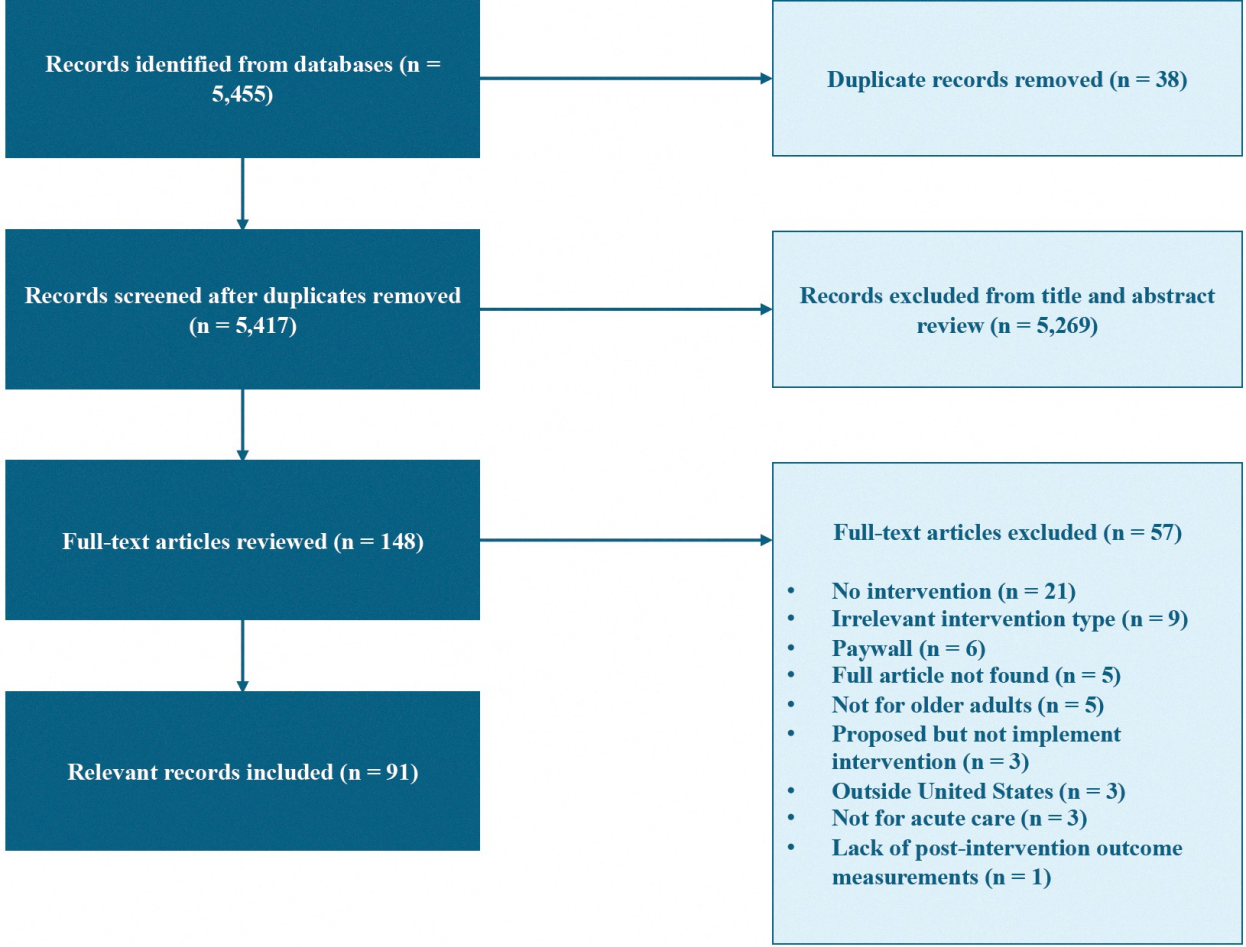
PRISMA flow diagram of the article selection process.

The database searches yielded a total of 5,455 abstracts (Fig 1): 5,084 from PubMed, 247 from Embase, and 124 from Scopus. After removing duplicates, 5,417 abstracts were screened, resulting in 148 articles identified for full-text screening. Of these, 91 articles met our selection criteria and were included in our review. We report characteristics of included studies (Table 1), followed by a classification of identified pre-discharge interventions and an analysis of author sentiment regarding intervention outcomes.

**Table 1:** Characteristics of the articles according to the eleven dimensions for analysis.

Authors and Year	Design	Intervention Description	Targeted Population	Intervention Motivation	Early Discharge Sentiment	Sentiment Reasoning
Ahmed et al., 2018 [98]	Retrospective Intent-to-Treat Analysis Study	Providing non-emergency radiology services on the weekends.	Patients who underwent an interventional radiology procedure on the weekends or intended to.	Reduce patient LOS and unnecessary admissions.	Positive	Reduced wait times for procedures and patient LOS.
Aicher et al., 2019 [115]	Pre-Post Study	Health system redesign.	Adult vascular surgery patients discharged while under the care of a vascular surgeon.	Reduce LOS given fixed payment for service insurance reimbursement processes.	Neutral	Intervention significantly reduced average LOS, but it was not associated with a financial benefit nor decrease in 30-day readmissions.
Alaraj et al., 2017 [123]	Pre-Post Study	Physician led multi-disciplinary huddles.	Subarachnoid hemorrhage patients (SAH).	Reduce LOS of patients with high risk of long LOS.	Positive	Reduced total hospital costs and mean LOS.
Allen et al., 2003 [45]	Pre-Post Study	Acute stroke unit (SU) based on the Acute Care for Elders (ACE) model of care.	Stroke and transient ischemic attack (TIA) patients.	Standardize essential elements that define a SU.	Neutral	Establishment of a SU improved survival and functional outcomes for patients with acute stroke but cost-effectiveness remains to be determined as well as whether benefits remain post-discharge.
Ansari et al., 2018 [113]	Retrospective Correlational Study	Reduction of LOS protocol.	Neurosurgical patients.	Evaluate whether reduced LOS would increase readmission rates.	Negative	A strong negative linear correlation was found between readmission rates and decreased hospital LOS.
Babb et al., 2017 [120]	Retrospective Observational Correlation Study	Oral nutritional supplements for heart failure inpatients.	Heart failure inpatients.	Improved nutrition to reduce LOS, costs, readmission, complication, and mortality	Neutral	Improved patient healing through better nutrition but required increases in patient LOS.
Bachman et al., 1987 [35]	Experimental Cohort Vs. Control Study	Geriatric acute care model, consisting of discharge planning initiated upon admission and team conferences with the inclusion of families.	Older patients age 65+.	The pressure for hospitals across the country to provide high-quality, cost-effective health care.	Positive	Reduced LOS and total hospital charges without decreasing quality of care.
Batlle et al., 2010 [68]	Retrospective Cohort Study	Early use of advanced imaging.	Inpatient admitted patients who had a CT, MRI or nuclear scintigraphy.	Reduce LOS.	Neutral	Significantly reduced LOS, but it is premature to suggest that clinicians should order advanced imaging studies early.
Beri et al., 2017 [79]	Retrospective Analysis Study	Using copeptin and cardiac troponin I (cTnI) biomarkers with a normal electrocardiogram at presentation and 2 hours after to identify candidates for early discharge with outpatient follow-up.	Adult patients presenting non-traumatic chest pain within 6 hours of symptoms onset for which an ED physician suspected acute coronary syndrome.	Reduce high costs of hospitalization among low-risk chest pain patients.	Neutral	The combination of a normal electrocardiogram at presentation with a normal troponin I and copeptin at presentation and after 2 hours defined a truly low risk population for MI or death at 180 days. However, author suggests further research should prospectively evaluate the management of these low-risk chest pain patients.
Carr et al., 2014 [121]	Observational Prospective Study	Health information exchange.	Emergency department patients whose physicians used the health information exchange and found relevant information.	Decrease health services use by emergency department patients.	Positive	Helped avoid use of duplicative radiology services.
Carr et al., 2018 [60]	Retrospective Pre-Post Study	Requiring target patients to receive highest level of trauma care.	70+ year old trauma patients.	Higher trauma activation is better care, but more costly.	Neutral	Helped provide better care attention lowering LOS and mortality but only for older adults of age 77 years or older.
Casale et al., 1998 [91]	Retrospective Correlational Study	Access to a specialist/physician with experience treating myocardial infarction patients.	Myocardial infarction patients.	Pushback on financial disincentive to see specialists for myocardial infractions.	Positive	Improved quality of care patients experienced.
Collier 1995 [36]	Cohort Study	Clinical care pathway with early discharge on the first postoperative day when feasible.	Elective carotid endarterectomy patients.	Improvement in quality management of carotid endarterectomy patients.	Negative	Increased presence of complications and ICU admissions which resulted in LOS increases, diminishing the cost efficiency of the carotid endarterectomy.
Crist et al., 1987 [104]	Randomized Controlled Trial	Therapeutic drug-monitoring program.	Acute care patients receiving aminoglycosides for more than 24 hours.	Decrease drug toxicity and hospital LOS associated costs.	Positive	Helped reduce unneeded extra drug therapy among patients.
Dalal et al., 2020 [111]	Pre-Post Study	Multimodal intervention encouraging use of nonopioid alternatives to reduce intravenous opioid use.	Inflammatory bowel disease hospitalized patients.	Opioid use in patients with inflammatory bowel disease (IBD) is associated with increased mortality.	Neutral	Reduced opioid exposure, LOS, and 30-day readmission rates. Additional research is needed to determine long-term benefits.
DeLa’O CM et al., 2014 [55]	Retrospective Pre-Post Study	Creation of the Geriatric Trauma Institute to promote quality care, reduce the length of stay, and reduce hospital charges.	Hospital patients of age 65+ years.	Need to promote quality care, reduce LOS, and reduce hospital charges.	Positive	Intervention was cost effective.
Eaton et al., 2019 [89]	Pre-Post Study	9-point risk assessment and Intravenous Antibiotics and Addiction Team (IVAT) for multidisciplinary care of persons who inject drugs (PWID).	Patients who inject drugs with acute infections (bloodstream infections, infective endocarditis (IE), skin and soft-tissue infections (SSTIs).	Reduce LOS, readmissions, and costs associated with acute infections among PWIDs with optimal management practices.	Neutral	Direct costs per patient admissions reduced by 33% despite higher costs incurred post-intervention but number of readmissions was not unchanged.
Ebinger et al., 2018 [86]	Retrospective 3-Phase Comparison Study	ZWOLLE Risk Score–a STEMI Risk Guided Triage Integrated Calculator	ST-segment-elevation myocardial infarction (STEMI) patients treated with percutaneous coronary intervention (PCI).	Increasing pressure to improve the value of healthcare at a lower cost.	Neutral	Findings highlight potential value of using the Zwolle Risk Score in STEMI to reduce costs, but author warns about the negative consequences related to the use of risk scoring tools.
Eron et al., 2001 [92]	Observational Case-Controlled Study	Use of infectious diseases (ID) hospitalists instead of an internal medicine (IM) hospitalist when discharging patients.	Hospital patients with community-acquired infections such as community-acquired pneumonia (CAP), urinary tract infection (UTI), and cellulitis.	Economic considerations have forced clinicians to consider strategies for earlier discharge from hospital.	Positive	Patients cared for by ID hospitalists had a shorter average length of stay with no associated increase in readmissions and increased patient satisfaction.
Farber et al., 2011 [52]	Retrospective Propensity Matched Case and Control Study	Mobile acute care for the elderly (ACE) (MACE) service for those cared for on a unit-based ACE service.	Inpatients of age 65+ years.	Improve functional outcomes without increasing hospital costs or LOS.	Positive	Mobile ACE service resulted in reduced LOS which lowered costs without impacting readmission or mortality rates.
Fishbane et al., 2007 [49]	3 Sequential Block Pre-Post Study	Standard order sets alone or with intensive clinical case management for CAP patients.	Patients admitted for community acquired pneumonia (CAP) with no admission in previous 30 days, not in ICU initially, no AIDs/cancer, not on immunosuppressant, not refusing antibiotics.	Reduce cost, reduce risk of nosocomial infections by reducing LOS.	Positive	Good case management helped reduced time from clinical stability to discharge.
Flarity et al., 2017 [59]	Comparative Study	2013 clinical practice guideline (CPG) for rib fractures, including monitoring of pulmonary function, early initiation of aggressive loco-regional analgesia, and early identification of deteriorating respiratory function.	Patients with traumatic rib fractures.	Lack of established national standard for rib fracture management.	Neutral	Helped limit narcotic usage, improve pulmonary function, and decrease LOS in most injured patients with chest trauma but patient safety was not evaluated.
Friedman et al., 2008 [50]	Cohort Study	Co-managed Geriatric Fracture Center program consisting of co-management, protocol-driven geriatric-focused care, and early discharge planning.	Older adults of age 60 years and above with a proximal, native, low impact, nonpathological femur fracture that received surgical repair.	Need to improve outcomes of care among older adult patients with hip fractures surgery.	Neutral	Helped reduce hospital readmissions, complications and mortality rates which increased hospital profits but more than 90% of the patients were discharged to a nursing facility.
Friedman et al., 2013 [109]	Retrospective Cohort Study	Triple anti-platelet drug therapy initiated 2 days after hospital admission or during emergency room visitation.	Acute coronary syndrome (ACS) adult patients.	Need to analyze the impact of triple therapy versus dual therapy on the more diverse ACS patient population.	Neutral	Triple anti-platelet therapy was associated with superior results but only for selected outcomes. Physicians need to carefully consider the risk-benefit ratio of triple anti-platelet therapy.
Gayed et al., 2013 [54]	Prospective Cohort Study	Lean Six Sigma process redesign for joint replacement patients.	Joint replacement patients.	Reduce LOS and reduce care costs.	Positive	Reduced LOS, eliminated need for non-VA care for patients undergoing total hip and knee replacement, and increased volume of total joint replacements.
Gheiler et al., 1999 [39]	Pre-Post Study	Clinical care pathway.	Radical prostatectomy patients.	Reduce LOS and care costs.	Positive	Helped reduce LOS which reduced hospital costs without increasing patients’ complications risk.
Gittell et al., 2000 [41]	Cross-Sectional Series Study	Relational coordination—management of task interdependencies.	Total hip and knee arthroplasty patients.	Improve quality of care and clinical outcomes while increasing efficiency.	Positive	Relational coordination was associated with improved quality of care, less pain, more functioning and shorter LOS.
Gould 2011 [96]	Randomized Controlled Trial	Discharge nursing intervention (DNI) aimed at promoting self-regulation of care for early discharge interventional cardiology patients.	Patients with cardiovascular disease (CVD) undergoing interventional revascularization procedures.	Little is known about how cardiovascular patients make sense of their condition and manage their care at home after receiving interventional procedures or medical management requiring shorter hospital stays.	Negative	At the end of the study, there was insufficient evidence to understand how early discharges impact post-treatment self-care among patients or the impact of the intervention on costs.
Gross 1995 [37]	Prospective Analysis Study	Early extubation program.	Cardiothoracic patients.	Changing trends and economic forces that emphasize reduced length of stay and early discharge have commanded the need for redesign in the perioperative management of cardiovascular surgical patients.	Neutral	Intervention caused modest cost reductions and preliminary findings demonstrate intervention is safe and effective only in a certain group of cardiovascular patients.
Hamdy et al., 2014 [118]	Retrospective Pre-Post Study	Glycemia-Targeted Specialized Nutrition (GTSN).	Diabetes mellitus (DM) patients.	Compare patient outcomes and costs for patients with diabetes mellitus receiving GTSN versus standard nutrition (STDN) formulas during acute care hospitalizations.	Neutral	GTSN was cost effective for patients but patient outcomes post-intervention or post-discharge were not evaluated.
Hastings et al., 2014 [56]	Prospective Cohort Study	Assisted early mobility for hospitalized older veterans (STRIDE) clinical demonstration program.	Hospitalized veterans aged 65 and older.	Address the urgent need for programs to promote early, safe mobility in hospitalized individuals to prevent negative consequences.	Neutral	Median LOS, 30-day ED visit and readmission rates were not significantly different from those enrolled in STRIDE versus not.
Hay et al., 1997 [64]	Prospective Controlled Time Series Cohort Study	Clinical practice guideline for LOS in the form of a 4-variable scoring system for initial risk assessment.	Upper gastrointestinal tract hemorrhage (UGIH) hospitalized patients.	Reduce hospital LOS while maintaining or improving quality of care.	Positive	Study was the first in its area to consider efficacy, safety, and acceptability of implementing a UGIH clinical LOS guideline which safely reduced hospital LOS.
Holland et al., 2003 [66]	Prospective Cohort Study	Probability of repeated admission screen to identify patients with nonroutine discharge planning needs.	Hospitalized older adults.	Need to identify patients who need more time/help for discharge planning earlier in their stay.	Neutral	The clinical utility of using the PRA as a screen for early identification of persons who use nonroutine discharge planning is very limited. Findings suggest STRIDE reduces risk for SNF care need for post-acute care.
Holland et al., 2013 [71]	Descriptive Cross-Sectional Cohort Study	Early Screen for Discharge Planning (ESDP)–an evidence-based discharge planning decision support tool	Hospitalized adults being discharged home.	Improve discharge planning to reduce readmission rates and unmet needs post-discharge.	Positive	The ESDP is effective as a decision support tool in identifying patients to prioritize for early discharge planning intervention.
Holland et al., 2017 [80]	Comparative Descriptive Survey Study	Early Screen for Discharge Planning (ESDP)–a decision-support tool.	Hospitalized adults.	Validate tool in regional hospital setting.	Positive	Findings were consisted with prior research and tool proved to be effective in a regional hospital setting.,
Horn et al., 1985 [103]	Prospective Pre-Post Study	Digoxin pharmacokinetic monitoring service.	Patients with a diagnosis of congestive heart failure (CHF), myocardial infarction (MI), or cardiac arrhythmia who are also prescribed digoxin.	Evaluate cost-effectiveness of clinical pharmacokinetics services (PKS) in hospitals on digoxin concentration which typically involve monitoring serum drug assay results, reviewing patient medical records, drug therapy consults with dosing recommendations, and patient drug therapy monitoring.	Neutral	Intervention was cost effective but impact on post-discharge patient outcomes were not followed up with.
Horowitz et al., 2002 [122]	Pre-Post Study	Provider education on reducing antibiotics for pneumonia care.	Pneumonia patients.	Improve patients’ knowledge of pneumonia care, reduce antibiotic use and LOS.	Positive	Intervention helped reduce time on antibiotics while increasing patient health knowledge.
Hou et al., 2008 [65]	Retrospective Cohort Study	Clinical assessment at admission to identify patients who require social work intervention for discharge planning.	Hospitalized patients admitted from their home.	Decrease patient LOS.	Neutral	Physician clinical assessments of patient function along with age may help to identify patients who have greater needs for social work interventions, reduce LOS, and decrease costs. However, additional prospective studies are needed.
Houck et al., 2004 [106]	Retrospective Non-Equivalent Cohort Vs. Control Group Study	Antibiotics administered within 4 hours of arrival.	Patients 65+ years of age with community acquired pneumonia.	Prevent patient deaths and reduce hospital costs.	Positive	Antibiotic administration within 4 hours of arrival can prevent deaths in the Medicare population, offers cost savings for hospitals, and is feasible for most patients.
Ichibori et al., 2019 [116]	Comparative Study	Next-day discharge (NDD) part of the very early (VED) discharge strategy.	Transcatheter aortic valve replacement (TAVR) adult patients.	Minimize healthcare system costs while optimally maintaining the patient’s functional capacity post TAVR.	Neutral	The VED strategy produced significantly shorted mean LOS, reduced hospitalization costs but may be hindered by complications and patient selection bias.
Jones et al., 2006 [47]	Cohort Study	Clinical pathway for inpatient management of atrial fibrillation.	Atrial fibrillation cardiology patients.	Test outcomes to adherence of the clinical pathway given discrepancies between expert consensus and community practice on established guidelines for atrial fibrillation patients.	Neutral	Patients requiring hospitalization for symptomatic atrial fibrillation had a nearly 50% reduction in LOS but with a trend towards increased utilization of risk-appropriate antithrombotic therapy. Root cause of shorter LOS is unknown (intervention versus cardiology care).
Kaboli et al., 2004 [93]	Prospective Quasi-experimental Study	Hospitalist usage.	Acute care patients admitted to the hospital.	Evaluate how the usage of hospitalists influences shorter LOS and costs.	Neutral	Patients managed by hospitalists had shorter LOS and lower costs than patients managed by nonhospitalists but had higher costs per day. No difference made on hospital readmissions.
Kandzari et al., 2003 [105]	Randomized 2 x 2 Factorial Controlled Study	Adjunctive abciximab after PCI as part of the Controlled Abciximab and Device Investigation to Lower Late Angioplasty Complications (CADILLAC) trial.	Myocardial infarction patients.	Economy push for the development of treatment strategies that provide both clinical benefit and mitigate costs.	Positive	Abciximab use with primary PCI in MI was associated with significantly reduced in-hospital ischemic adverse events and hospital stays, which translated into a ~70% offset of the cost of abciximab therapy.
Kates et al., 2011 [53]	Retrospective Cohort Study	Comanaged protocol driving fracture management program.	Patients of age 60+ years with native, non-pathologic hip fracture.	Improve hospital profitability.	Positive	Helped reduce costs without worsening patient mortality, complications or readmission risks.
Keyes et al., 2014 [57]	Pre-Post Study	New dedicated Senior emergency department.	65 years of age and older patients.	Reduce recidivism.	Neutral	A new senior ED was not associated with reduced ED recidivism or hospital LOS but was associated with decreased rate of admission.
Kontos et al., 2003 [67]	Cohort Vs. Control Study	Risk-based triage system for the evaluation of chest pain using early myocardial perfusion imaging (MPI).	Patients with chest pain complaints.	Need to improve diagnostic sensitivity of chest pain present in patients due to costs, inappropriate discharges and mortality rates.	Neutral	Mean costs per encountered were reduced for each triage level patient due to diagnostic protocol but angiography rates and LOS were only reduced for low-risk patients.
Kozma et al., 2010 [34]	Descriptive Retrospective Analysis Study	Reducing hospital LOS by 1 day protocol.	Hospitalized patients with community acquired pneumonia.	As a component of healthcare reform, payers, hospital administrators, and physicians are looking for ways to reduce hospital expenditures and improve efficiency.	Neutral	Cost effective if tests and treatments are compressed with shorter LOS. Otherwise, a shorter LOS caused an unsafe discharge by reducing patient healing process time.
Kucenic et al., 2000 [42]	Pre-Post Study	Clinical pathway for myocardial infarctions patients.	Myocardial infarction patients.	Implementing evidenced based practice to have uniformity of care and reduce costs.	Positive	Pathway reduced LOS and hospital charges without compromising care quality and outcomes.
Kupensky et al., 2015 [58]	Retrospective Correlational Study	Palliative medicine consultations.	Patients of age 65+ years admitted to trauma services.	Reduce LOS.	Positive	Early PMC was associated with reduced LOS.
Lee et al., 1999 [40]	Cohort Vs. Control Study	Early extubation and fast track treatment protocols.	Elderly coronary bypass surgery patients	Determine whether fast-track recovery and cost containment in an elderly population can be replicated under an elderly population undergoing isolated coronary bypass operations.	Neutral	Accelerated discharge was deemed safe and feasible but only in 34% of the older population.
Levine et al., 2018 [99]	Retrospective Pre-Post Study	Use of a medical toxicology service (MTS).	Rattlesnake envenomation patients.	Cases of envenomation rarely involve medical toxicologists.	Neutral	Intervention reduced LOS by a full day which was a major contributor for cost-effective care, but hospital readmissions were not reduced.
Mahler et al., 2013 [72]	Secondary Analysis of Clinical Trial	Unstructured clinician assessment, North American Chest Pain Rule (NACPR), and HEART score with serial troponin measures.	Patients with symptoms of suspected acute coronary syndrome (ACS), starting within 6 hours of presentation and lasting for at least 30 minutes, in whom the physician planned objective cardiac testing.	Thrombosis in Myocardial Infarction (TIMI) risk score and Global Registry of Acute Coronary Events (GRACE) scores are recommended to aid risk stratification, but they are not sensitive enough to avoid objective testing or inpatient care.	Positive	The HEART score with 0- and 3-hour serial troponin measures identifies a substantial number of patients for early discharge while maintaining high sensitivity for acute coronary syndrome.
Mahler et al., 2015 [74]	Secondary Analysis	Accelerated Diagnostic Protocol (ADP) for early emergency department discharge given chest pain.	30+ year old patients symptomatic of acute coronary syndrome	Validate decision rule used to free up hospital bed space.	Positive	Intervention allowed for the efficient allocation of resources for patients which helped discharge patients safely.
Mahler et al., 2015 [75]	Randomized Control Trial	HEART Pathway which identifies emergency department patients with acute chest pain for early discharge.	Adult emergency department patients with acute coronary syndrome without ST-elevation on electrocardiogram.	Reduce diagnostic waste on low-risk patients which should save time and money.	Positive	Intervention was associated with a reduction in resources utilization without driving major adverse cardiac events to occur.
Mahler et al., 2016 [78]	Secondary Analysis of Randomized Controlled Trial	Accelerated Diagnostic Protocols (ADPs).	Patients presenting with symptoms suggestive of acute coronary syndrome (ACS).	Determine how provider nonadherence may threaten the safety and effectiveness of ADPs.	Neutral	Under and over testing occurred due to nonadherence to intervention which caused a reduction in safe early discharges by 13% but without MACE presence among patients within 30 days.
Mansouri et al., 2011 [108]	Retrospective Pre-Post Study	Infectious disease team had to approve dispense of any ordered restricted antibiotics.	New community acquired pneumonia patients with no recent use 4of antimicrobials and no ICU use.	Reduce overuse of restricted antibiotics class, reduce costs and LOS.	Positive	Helped reduce use of restricted antibiotics, costs and LOS.
Mayo et al., 1996 [38]	Pre-Post Study	Quality improvement of process of care.	Patients admitted to hospital with acute asthma exacerbation for 1–10 days.	Improve care quality.	Positive	LOS was reduced without increasing readmission rates.
Melamed et al., 2020 [100]	Retrospective Cohort Study	Consensus-based treatment algorithm (TA) and pulmonary embolism response team (PERT).	Patients with submassive and massive pulmonary embolism (PE).	Address the challenges faced by the management of patients with submassive and massive pulmonary embolism when adverse outcomes are prevalent during treatment.	Neutral	Intervention helped improve in hospital care efficiency, but mortality rates were unchanged.
Meneghini et al., 2017 [81]	Retrospective Cohort Study	Patients with sufficiently high scores on assessment offered outpatient surgery with same day discharge.	Patients who underwent unilateral primary total joint arthroplasty by a single surgeon.	Cost control, operating room control, and standardization of patient selection for outpatient treatment.	Neutral	The Outpatient Arthroplasty Risk Assessment [OARA) score for primary total joint arthroplasty has more precise predictability than other scores for the same or next day discharge and is enhanced with a robust patient education program. However clinical program maturity before adoption is advised.
Mundy et al., 2003 [46]	Randomized Clinical Trial	Early mobilization [EM).	Hospitalized adults with community-acquired pneumonia.	Determine whether EM of patients with CAP during the first day of hospitalization and progressive mobilization each subsequent day safely shortens LOS.	Positive	Enhancing nursing focus on patients helped reduce LOS without increasing risk of adverse outcomes.
Naughton et al., 1994 [90]	Randomized Controlled Trial	Assigning geriatrician and social worker as primary managing team.	Adults of age 70+ years admitted from emergency department to inpatient (non-ICU) when no internist was on staff.	Reduce costs.	Positive	Reduced costs without noticeable differences in patient outcomes.
Ni et al., 1999 [112]	Post-Intervention Multivariate Analysis Study	Hospital controlling hospitalization rate and shortening length of stay.	Congestive heart failure (CHF) patients within the aging population.	Lack of data regarding the effectiveness of the intervention aimed at controlling the cost of care for patients with congestive heart failure.	Neutral	Hospitals were able to reduce hospitalization and resource utilization costs but there was an increase in patients discharged to skilled nursing facilities and home health services, a decrease in patients discharged home, a decrease in hospital admissions for patients of age 65+ years and increase in hospital-mortality rates.
Nyswonger et al., 1992 [117]	Retrospective Cohort Study	Early enteral nutrition.	Geriatric hospitalized nonsurgical stroke patients.	Today’s milieu of cost containment and limited reimbursement requires caregivers to look for ways to shorten the length of hospital stay and association between malnutrition and strokes.	Neutral	Although a shorter LOS was observed among stroke patients, further studies are needed to determine whether early enteral nutrition drives shorter LOS.
Padula et al., 2009 [51]	Nonequivalent Cohort Vs. Control Study	Nurse-driven mobility protocol (remove mobility barriers such as catheters, walk patients 3–4 times per day, assist with toileting/chair and bed transfer).	Patients of age 60+ yeas with a length of stay > = 3 days, fluent in English, free of severe physical impairment and cognitively intact.	Reduce functional decline and LOS.	Positive	Intervention help improve mobility among treated patients.
Peralta et al., 2020 [101]	Pre-Post Study	Hospital-wide quality improvement process through a care delivery redesign (CDR).	Vascular surgery patients	Improve patient care efficiency, clinical documentation, and reduce LOS for improved revenue.	Positive	CDR decreased LOS and improved clinical documentation which reduced costs and increased hospital revenue.
Perry et al., 2020 [102]	Retrospective Cohort Study	Multiprofessional acute trauma health care (mPATH) team based on Lean methodologies.	Severe traumatic brain injury (TBI) and spinal cord injury (SCI) patients older than 55 years.	Nationwide drive to reduce LOS, improve quality and delivery of care in a cost-efficient manner.	Positive	mPATH decreased LOS and cost savings without sacrificing mortality or readmission rates.
Reddy et al., 2001 [43]	Retrospective Cohort vs. Control Study	Clinical practice guideline for community-acquired pneumonia (CAP) written by a multidisciplinary panel of UCSF clinicians and largely based on the American Thoracic Society guideline.	General medical service patients both from regular medical floors and intensive-care units (ICU) and patients with a diagnosis of CAP.	Compare the clinical practice guideline versus the hospitalist system implemented at UCSF in 1995.	Neutral	Intervention significantly lowered 10-day readmission rates, reduced costs and LOS of patients diagnosed with CAP but not among other patients.
Reed et al., 2004 [94]	Pre-Post Study	LOS Officer (In charge of communicating discharge planning with family, coordinates outpatient services and meets with hospital staff).	Adult vascular surgery patients with aortic aneurysm, carotid artery stenosis, lower extremity critical ischemia or amputation, and foot debridement.	Optimize use of hospital resources.	Positive	Reduced LOS without being associated to patient adverse outcomes, resulting in cost savings for hospital.
Riley et al., 2017 [82]	Randomized Controlled Trial	HEART Pathway.	Acute coronary syndrome (ACS) emergency department patients.	Evaluate the impact of the Heart Pathway on the cost of care, which has not been previously reported.	Positive	Heart Pathway significantly lowered cost per individual, reduced cardiac testing, increased rates of early discharge, and optimized resource utilization without notable increased risks for adverse outcomes.
Rittenhouse et al., 2015 [76]	Pre-Post Study	AntiCoagulation and Trauma (ACT) Alert which screens patients of age 65+ years on anticoagulation using Glasgow Coma Score, required assessment, CT scan, and normalized INR within 30 minutes.	Patients of age 65+ years on anticoagulation sustaining minor injuries.	Reduce negative consequences of triage for elderly patients on anticoagulation with minor injuries.	Positive	ACT Alert improved emergency department throughput and reduced hospital LOS while effectively identifying at-risk mildly head injured geriatric patients on anticoagulation.
Rodriguez-Araujo et al., 2018 [114]	Secondary Analysis Study	Same-day discharge policy.	Patients undergoing elective transradial percutaneous coronary intervention (TR-PCI).	Clinical and financial push to discharge early patients undergoing TR-PCI.	Positive	Same-day discharge seemed to be a safe and feasible clinical practice, with significant potential savings for the US healthcare system.
Rothberg et al., 2010 [107]	Retrospective Cohort Study	Early antibiotic treatment according to a COPD treatment guideline.	Acute exacerbation, emphysema or respiratory failure patients with pre-existing COPD.	Further evaluate use of early antibiotic treatment among patients hospitalized with COPD to generate current evidence.	Neutral	Early antibiotic treated patients were less likely to incur other procedures, had a lower incidence of treatment failure, lower in-hospital mortality risk and costs but had slightly longer LOS and higher readmissions for diarrhea.
Rozell et al., 2017 [83]	Training and Validation Sample Study	Early discharge following total hip replacement or total knee replacement and development of complications risk model.	Elective surgery total hip/knee replacement patients.	Risk model motivated by improved pain management and early mobilization protocols pushing for early discharge.	Negative	Patients with cirrhosis, congestive heart failure, or chronic kidney disease should be excluded from early discharge total joint arthroplasty protocols as they are more likely to experience complications and longer LOS. Complications risk model predicted only moderately.
Rudolph et al., 2014 [73]	Quality Improvement Project Study	Delirium Toolbox Intervention.	65 years of age or older adult patients.	Prevention of delirium due its high prevalence rates among hospitalized patients and its association to increased morbidity and mortality.	Positive	Patients who received delirium risk modification items had an associated shorter LOS, lower rates of restraint use, and lower variable costs. Intervention also improved medical record documentation regarding delirium and hospital experiences specifically for patients at intermediate risk for developing delirium.
Shah et al., 2012 [70]	Retrospective Data Analysis Study	Thrombolysis in Myocardial Infarction (TIMI) score tool use in cost-effectiveness of chest pain observation units (CPOUs).	Patients with unstable angina and acute coronary syndrome.	Evaluate the use of TIMI which is a simple and easy tool to use in chest pain observation units (CPOUs) whose impact has not been thoroughly studied previously.	Positive	Cost effective intervention.
Sharkawi et al., 2017 [84]	Retrospective Analysis Study	CADILLAC risk score.	Patients with ST-elevated myocardial infarction (STEMI).	Evaluate CADILLAC risk score’s effectiveness in patient stratification for early discharge relative to patient safety.	Neutral	Intervention was useful among low-risk patients but not among moderate to high risk patients.
Shilian et al., 2020 [63]	Retrospective Investigation Study	Daily integrated conferences (ICCs).	Patients with chronic obstructive pulmonary disease (COPD).	Inefficiencies in care coordination often led to unnecessary increase in length of hospital stay.	Positive	Helped decrease costs and LOS while improving coordinated care.
Slauenwhite et al., 1998 [33]	Pre-Post Study	Early stay care plan development, delivery of care, follow up and plan correction (Enhanced Early Discharge).	Adults of age greater than 60 years with hip fracture living in the community.	Reduce LOS and promote independent living	Positive	LOS was significantly reduced while patients were satisfied with program health outcomes.
Snider et al., 2015 [119]	Comparative Study	Oral nutrition supplementation (ONS).	Medicare patients with COPD.	Evaluate the impact of ONS on readmission risk, LOS, and cost among hospitalized COPD patients which are at a high risk of nutritional deficiency.	Neutral	Although ONS was cost effective for both the hospital and Medicare, benefits of ONS overall were mixed.
Somanchi et al., 2011 [69]	Basic Stepped Wedge Study	Potential nutrition risk screen criteria at admission form to identify at risk patients. Daily nutrition assessment after.	Adults admitted to two medical wards in same hospital.	Malnutrition has been linked with longer LOS, morbidity, and mortality.	Positive	Proper nutrition shortened LOS, increasing hospital savings.
Soto et al., 2018 [61]	Cohort Study	HEART TRACKS–a structural transitional care pathway.	Patients presenting with cardiac-related complaints.	To improve care-coordination following an ED visit.	Neutral	Structured transitional care pathway can be effective in shifting care delivery from hospital-based to lower cost ambulatory settings but only at large institutions.
Southern et al., 2007 [95]	Cohort Vs. Control Study	Increased employment of hospitalists.	Patients with specific diagnoses like asthma, COPD, coronary disease, diabetes, etc.	To determine whether patients with specific diagnoses or discharge needs account for the association between hospitalist care and reduced length of stay.	Neutral	Early discharge was only beneficial among patients with conditions requiring specialized services after discharge.
Stopyra et al., 2015 [77]	Secondary Analysis Study	Emergency department assessment of chest pain score using an accelerated diagnostic protocol to assess patients for suitability of early discharge.	Emergency department patients with symptoms of acute coronary syndrome.	More efficient allocation of resources.	Positive	Assessment was noted to have a high sensitivity for major adverse cardiac events, enabling appropriate early discharges.
Stopyra et al., 2017 [85]	Secondary Analysis of Randomized Control Trial	HEART Pathway to help identify emergency department patients with acute chest who don’t need objective testing.	Emergency department patients with symptoms of acute coronary syndrome.	Reduce use of hospital resources.	Positive	Reduced hospital resources utilization such that the intervention was cost-effective.
Tesson et al., 2018 [87]	Retrospective Cohort Study	HEART Pathway risk prediction tool.	Patients with chief complain of chest pain who were referred to the CDU for further care.	Determine usefulness of the HEART Pathway in a U.S. hospital and calculate miss rate.	Neutral	Applying HEART Pathway missed 1.2% of patients with MACE. Can identify patients at low risk for MACE but risk is not homogenous within population.
Treat et al., 2016 [110]	Nonrandomized Study	Standard of care protocol with the addition of sulfate-free polyethylene glycol electrolyte lavage solution (PEG).	End-stage renal disease adult patients undergoing renal transplantation.	Reduce prolonged hospitalization and costs after kidney transplantation.	Positive	Using PEG solution enables lower hospitalization costs with ease of administration that is well tolerated and has minimal side effects.
Walsh et al., 2001 [44]	Difference In Difference Randomized Controlled Trial	Nurse case manage modifying care pathway.	Patients undergoing infrainguinal bypass surgery.	Reduce LOS.	Positive	Intervention reduced institutional deviations (inefficiencies) from desired care pathway.
Walsh et al., 2018 [88]	Retrospective Pre-Post Study	Implementation of PCT testing to determine use of antibiotic therapy.	Patients with pneumonia.	Build evidence for Procalcitonin guidance to treat Pneumonia, reduce antibiotic use and LOS.	Positive	Intervention allowed for more informed antibiotic therapy leading to appropriate ending of medications and earlier discharge of patients.
Weems et al., 2019 [62]	Pre-Post Study	Serial measurement and feedback adaption for care standardization.	Heart failure and pneumonia patients.	Improve quality of care for patients and reduce unnecessary costs.	Positive	Reduced care variation and improved overall quality of care scores individuals provided. Decreased LOS and readmissions leading to major savings.
Zemencuk et al., 2006 [48]	Pre-Post Study with Non-Equivalent Control Group	Physicians were told their patients’ LOS would be compared to peers.	General patient population at several VA hospitals.	Assess if physician knowledge of LOS ranges reduces LOS.	Positive	Helped reduce LOS without adversely affecting physician satisfaction.
Zhu et al., 2016 [97]	Retrospective Comparative Study	Nurse practitioner (NP)-based chest pain unit.	Chest pain hospital patients.	Evaluate efficacy and outcomes of a pathway-based, NP-run telemetry unit in treating patients admitted to large hospitals with chest pain.	Neutral	Average LOS was shorter for patients in the NP unit as well as 90-day readmission rates, but further evaluation is needed.

### Characteristics of included studies

**Fig 2:**
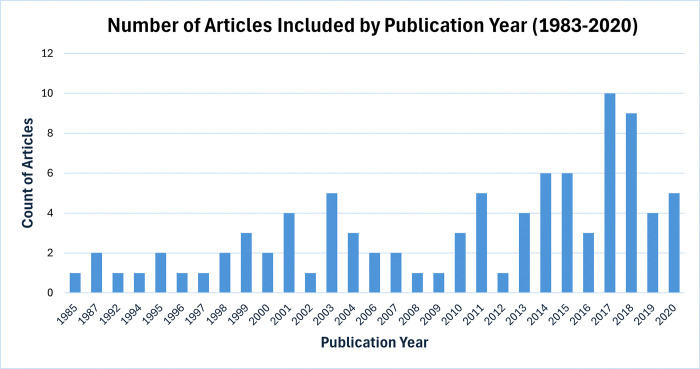
Distribution of articles by publication year.

Our sample included 91 primary research articles. Charted characteristics of the included articles are displayed in Table 1 (see S1 Table for detailed charted characteristics of the study populations), while S2 Table presents the charted measured outcomes and quantitative results of the interventions. The dominant study design observed was pre-post studies (n = 24), followed by cohort-based studies (n = 19), analysis-based studies (n = 13), randomized controlled trials (n = 8), cohort vs. control studies (n = 8), comparison-based studies (n = 6), correlational studies (n = 3), and observational studies (n = 3). The remainder varied among training and validation, cross-sectional, quasi-experimental, basic stepped wedge, investigation, nonrandomized, and quality improvement project-based studies (n = 1, respectively). The distribution of articles per publication year is illustrated in Fig 2. Over half of the research studies (n = 56/92) were published in the last 10 years of the search period.

The most common patient populations targeted were those with heart diseases or undergoing heart surgery procedures and treatments (n = 32) and those with respiratory diseases or community acquired pulmonary infections (n = 19), including pneumonia and chronic obstructive pulmonary disease (COPD). Some studies targeted older adults of different ages (n = 16) or patients with hip or knee joint fractures (n = 8). The remaining studies focused on patients during their post-surgery recovery (n = 4), undergoing radiology procedures (n = 2), with gastrointestinal diseases (n = 2), specifically being in the emergency department (ED) (n = 2), treated with routine specific medication treatment (n = 2), diabetes mellitus (n = 1), brain injuries (n = 1), or envenomization (n = 1). Patient sample sizes varied from 23 patients to 1,471,295 patients [33, 34].

### Classification of interventions

We identified eight categories to classify pre-discharge interventions: *clinical management*, *diagnostic/risk assessment tools*, *staffing enhancements*, *drug administration*, *LOS protocols*, *nutrition planning*, *and communication improvements*. For each category, we provide a definition and examples.

### Clinical management

Of the 87 distinct pre-discharge interventions identified among the 91 primary studies included in this scoping review, 30 (34%) were classified under the category of clinical management [33, 35–63]. This category includes pre-discharge interventions involving the development and implementation of clinical practice guidelines, pathways, programs, care standardizations, or care units for specific patient populations. For example, Collier (1995) conducted a 3.5-year cohort study to determine the effects of a clinical pathway for patients undergoing elective carotid endarterectomy, which provided patient education and early discharge in the first postoperative day [36]. Gross (1995) conducted a prospective analysis of patient interviews and medical records to determine the safety and efficacy of early extubation as a redesigned perioperative management approach for cardiovascular surgical patients [37]. Bachman et al. (1987) analyzed an implemented geriatric acute care model with specialized discharge planning initiated upon admission, which included care team conferences with patients’ families [35]. Reddy et al. (2001) compared the impact of using the new community-acquired pneumonia (CAP) clinical practice guideline against the care provided to patients via the hospitalist system used by the University of California San Francisco Medical Center since 1995 [43].

In more recent investigations, Soto et al. (2018) developed HEART TRACKS—a structured transitional care pathway for patients presenting with cardiac-related complaints—to improve care-coordination for appropriate testing and follow-up appointments with a cardiologist after an ED visit [61]. Weems et al. (2019) performed a pre-post analysis of clinical practice, patient outcomes, and costs to determine if standardizing care provided by hospitalists for heart failure and pneumonia patients at Novant Health reduced intrafacility care variation [62]. Shilian et al. (2020) conducted a retrospective investigation at two Ohio osteopathic community hospitals on the use of daily integrated care conferences (ICCs) meant to improve care coordination of COPD patients [63].

### Diagnostic/risk assessment tools

Diagnostic protocols, screening tools, risk assessments, triage system adjustments, risk stratifying tools, and risk scoring algorithms defined the category of diagnostic/risk assessment tools, which consisted of 22 (25%) different interventions identified in this review [64–89]. Four distinct studies discussed the same intervention, the HEART Pathway [74, 82, 85, 87], and two others discussed the Early Screen for Discharge Planning (ESDP) risk stratifying tool [71, 80]. We found that these tools were available either as fillable hand sheets and computerized software or interfaces. This category was predominantly associated more with publications from 2001–2020. The only pre-2000s intervention in this category is of Hay et al., 1997 where a retrospectively validated scoring system using four independent variables (hemodynamics, time from bleeding, comorbidity, and esophagogastroduodenoscopy (EGD)) was used to predict risk of adverse events and identify patients potentially suitable for early hospital discharge [64].

Interventions developed and implemented at the beginning of the 21^st^ century mainly focused on improving the discharge planning process and reducing discharge delays that may contribute to longer LOS. In 2001, Hou et al., studied the usage of clinical assessments to identify patients who may benefit from a social worker’s help to expedite the discharge planning process and reduce the overall hospitalization LOS of patients [65]. Similarly, Holland et al., in 2003 used a Probability of Repeated Admission (PRA) screening tool to identify patients that would incur a nontypical discharge process by requiring a new referral for services, to improve their discharge planning. In 2013, Holland et al., also created and applied a distinctive early screen for discharge planning (ESDP) risk stratifying tool to aid in the decision making of hospital discharging and identification of patients that would need or require more time allocated to the discharge planning phase [71]. The ESDP risk stratifying tool was later on integrated at a regional hospital to verify that it performed as expected even with a different population and setting in 2017 [80].

Remarkably, fourteen different studies targeted patients with chest pain and cardiovascular conditions such as acute myocardial infarctions (AMI) [67, 70, 72, 74–79, 82, 84–87]. In 2003, Kontos et al., determined the cost-effectiveness of a comprehensive, risk-based triage system that used early myocardial perfusion imaging (MPI) [67]. Shah et al.’s 2012 study examined the impact of using the Thrombolysis in Myocardial Infarction (TIMI) risk stratification scoring model in chest pain observation units (CPOUs) of EDs among patients admitted [70]. Mahler et al. (2015) developed and tested a 2-hour accelerated diagnostic protocol (ADP) to help identify chest pain patients for early discharge [75]. Another example of an ADP for chest pain patients is the emergency department assessment of chest pain score accelerated diagnostic protocol (EDACS-ADP) created by Stopyra et al. (2015) to improve risk stratification of patients [77]. Only two other studies focused on a patient population besides those with chest pain cardiovascular conditions. Specifically Meneghini et al. (2017) and Rozell et al. (2017) calculated and assessed the risk score for potential complications in post-arthroplasty patients using risk assessment algorithms [81, 83].

Rather than focusing on specific patient populations, several studies concentrated on developing risk assessments for various health concerns. These assessments aimed to measure the potential risk of a patients incurring malnutrition [69], developing delirium [73], or using certain drugs [89]. Notably, some risk assessment interventions, such as the HEART Pathway [75], the Controlled Abciximab and Device Investigation to Lower Late Angioplasty Complications (CADILLAC) risk score [84], and the Zwolle Risk Score (ZRS) [86], were developed to serve dual purposes:

1. Evaluate the likelihood of complications and adverse events in their respective patient populations.
2. Use the risk scores as proxies for early discharge.

In all three cases, this dual functionality of the risk assessment tools is a key feature that distinguishes them from others and enables clinicians to make decisions regarding patient disposition and resource allocation. This dual functionality also underscores the value of these risk assessment tools in enhancing clinical decision-making and optimizing patient care pathways.

### Hospital staffing enhancements

Thirteen (15%) interventions in this category involved changes in hospital staffing [90–102]. These included increased employment of hospitalists, dedicated use of nurse practitioners or hospitalists, improved access to a specialist/physician, additional use of social workers for specific care management, incorporation of new care roles or multidisciplinary care teams, and extensions of medical service times in hospitals. The earliest published intervention in this category was in 1994 by Naughton et al., who assigned a geriatrician and social worker as the primary management team for the care of older adult patients, reducing LOS by 2.1 days [90]. Similarly, Southern et al. (2007) evaluated the impact of a new hospitalist model of care for inpatients requiring complex discharge planning and close monitoring. This model assigned patients to hospitalists rather than traditional attending physicians, aiming to minimize subspecialist consultations and coordinate timely discharge plans [95]. A more recent example of having dedicated hospital staffing is the 2016 study by Zhu et al., which examined studied the effectiveness of using a nurse practitioner (NP)-run unit for evaluating patients with chest pain. The intervention proved effective, increasing the use of diagnostic testing and reduced LOS by an average of 2.7 days [97].

More recent studies published in 2020 focused on the usage of multidisciplinary care teams. Melamed et al. (2020) evaluated the use of a multidisciplinary pulmonary embolism response team (PERT) and a consensus-based treatment algorithm (TA) [100]. The TA was designed to guide the PERT in selecting and discussing a single treatment per patient, based on existing guidelines, current publications, and the treatment center’s expertise and resources [100]. Peralta et al. assessed the implementation of a care delivery redesign (CDR) multidisciplinary team [101]. This team included the Chief of Vascular Surgery, an inpatient NP, a dedicated case manager, a clinical documentation improvement specialist, and vascular surgery residents and faculty. The NP facilitated patient care coordination, resident system-based education, and multidisciplinary collaboration. Similarly, Perry et al. studied the impact of a dedicated multiprofessional acute trauma health care (mPATH) team on the LOS of patients with severe traumatic brain and spinal cord injuries [102]. The mPATH team comprised of a physical, occupation, speech, and respiratory therapists, a nurse navigator, a social worker, an advance care provider, and a physician.

### Drug administration

Pharmaceutical-related interventions also played a significant role in reducing hospital LOS, accounting for 10% (9) of the interventions reviewed [103–111]. This category encompasses a range of strategies, including the use of alternative drug treatments, changes in medication timing, revisions to drug treatment approval processes, and implementation of therapeutic drug monitoring (TDM) programs.

The earliest published interventions in this category relate to TDM programs. Horn et al. (1985) focused on a pharmacist-led pharmacokinetic monitoring service (PKS) for digoxin, a medication with a narrow therapeutic index, to improve patient dosing and lead to shorter hospital stays [103]. Similarly, Crist et al. (1987) implemented a TDM program for aminoglycoside antibiotics to assess its impact on total dose administered and reduce hospitalization costs [104]. Building on these approaches, Mansouri et al. (2011) later introduced an antibiotic restriction program (ARP) intervention. While sharing the goal of optimizing pharmaceutical use, the ARP focused more on regulating the prescription of antibiotics for CAP by enforcing approval prior to the dispensing of any restricted antibiotics such as piperacillin/tazobactam, cefepime, and ertapenem [108].

### LOS protocols

Six interventions (7%) in this category focused exclusively on hospital policies mandating patient LOS to be reduced by at least one day [34, 112–116]. Ni et al., (1999) demonstrated that for patients with congestive heart failure, hospital policies and management strategies significantly shortened LOS. This reduction was accompanied by an increase in discharges to skilled nursing facilities (SNFs) and home health services, ensuring continued post-discharge care [112]. Similarly, Kozma et al. (2010) implemented a 1-day reduction policy for patients with CAP, aiming to achieve economic benefits in response to healthcare reforms targeting reduced hospital expenditures [34].

### Nutrition planning

This category encompassed specialized nutrition programs, alternative methods for delivering nutrition, and oral nutritional supplements, accounting for four interventions (5%) [117–120]. Nyswonger and Helmchen (1992) focused on the use of enteral nutrition or tube feeding [117]. More recent studies, such as those by Snider et al. (2015) and Babb and Rohrer (2017) examined the effects of oral nutrition supplementation among patients with COPD and heart failure patients [119, 120].

### Communication improvements

Interventions aimed at improving communication between healthcare providers, between providers and their patients, or between a hospital and other post-acute care services comprised this category, which included three (3%) interventions [121–123]. An example is the study by Horowitz and Chassin (2002), which enforced provider education on antibiotics to enhance patient knowledge and reduce antibiotic usage in the treatment of pneumonia [122]. These interventions highlight the importance of effective communication in optimizing patient care.

### Intervention drivers

Motivations driving the development and implementation of each intervention can be found in Table 1. Common motivations included the need to reduce healthcare costs or increase hospital profitability, either directly or through resource optimization (n = 35), improve, standardize, or validate care protocols or tools (n = 35), and decrease LOS (n = 32). Other common motivational drivers included the desire to reduce mortality (n = 5), hospital readmissions (n = 4), or post-procedural complications (n = 2).

### Author sentiment on reported outcomes

Frequently measured outcomes to evaluate the intervention’s effectiveness or efficiency included patient LOS (n = 72), hospital readmissions over varying time frames (n = 31), hospitalization costs (n = 26), mortality (n = 22), post procedural adverse events or complications (n = 20), and discharge destination or disposition to a specific post-acute care facility (n = 20) (S2 Table). Additional measures included quality of care, time to treatment, physician adherence to risk assessment tool, care service utilization. Notably, only two studies measured patient satisfaction while one determined physician satisfaction.

Author sentiment regarding the impact of early discharge interventions was generally positive (53%, n = 48). For instance, Crist et al. (1987) viewed the implementation of a therapeutic drug-monitoring program favorably, noting reductions in unnecessary additional drug therapy among patients [104]. Hay et al. (1997) demonstrated the efficacy of a risk stratification scoring system for UGIH, which safely reduced hospital LOS for selected low-risk patients. Their study was pioneering in considering the efficacy, safety, and acceptability of implementing a UGIH LOS guideline [64]. In more recent years, Ahmed et al. (2018) highlighted how providing non-emergency radiology services on the weekends helped reduce patient LOS and unnecessary hospital admissions [98]. Similarly, Rodriguez-Arajo et al. (2018) reported that a same-day discharge policy was cost-savings effective with no difference in readmission or mortality rates [114].

Neutral author sentiment (mixed outcomes) was noted in 43% of interventions (n = 39). For instance, Lee et al. (1999) regarded that the intervention of early extubating along with the fast-track treatment protocol in elderly patients was safe and feasible but only in 34% of the older population [40]. Mahler et al. (2015) observed that the usage of ADPs for chest pain was associated with no major adverse cardiac events within 30 days but due to intervention nonadherence, safe early discharge decreased by 13% [75].

Negative author sentiment was described in the remaining 4% of interventions (n = 4). The study of Collier (1995) describes that complications and admissions to the ICU were observed because of the new clinical care pathway which in turn increased patients’ LOS and costs [36]. More recently, Ansari et al. (2018) observed that a strict hospital policy to reduce patient LOS among hospitalized neurosurgical patients was associated with an increased readmission rate, increasing care costs for patients and reimbursement penalties for the hospital [113].

## Discussion

In this scoping review, we analyzed 91 articles published between 1985 and 2020 that focused on pre-discharge interventions aiming at decreasing LOS among older adults in acute care settings. Our findings suggest that pre-discharge hospital interventions are increasingly used to promote early hospital discharge of older adults in acute care older settings in the US under the PPS. Furthermore, our literature synthesis builds on previous reviews of early discharge interventions by providing a broader understanding of intervention characteristics and author sentiment regarding their impact.

To address the type of interventions that exist, we identified eight different categories of pre-discharge interventions, expanding both in number and scope those identified in previous reviews. Unlike Coffey et al. (2019) and Siddique et al. (2021), our scoping review exclusively focused on interventions developed for and implemented in older adults from the time of admission to an acute care setting up to the discharge planning phase [27, 28]. By concentrating on older adults, we aim to understand the impact of interventions on a commonly hospitalized at-risk population, characterized by a wide range of cognitive and functional health states. This targeted approach provides valuable insights for enhancing care, especially considering the projected increase in the US older adult population to 94.7 million by 2060 [124]. Focusing on interventions before the discharge planning phase allows us to compare and analyze the effects experienced by patients resulting from the modified care they receive within a hospital. Additionally, this approach also enables us to evaluate hospital resource utilization for cost-analysis purposes.

It is worth noting that clinical management changes, particularly quality-improvement initiatives, were the most commonly observed category, a finding also emphasized by Coffey et al. (2019) [27]. Our review also identified the use of diagnostic and risk assessment tools as a growing type of pre-discharge intervention. These tools, broadly known as computerized clinical decision support systems (CDSSs), help provide healthcare professionals with valuable information to aid in patient care. Technological advancements, including the use of electronic medical records and advanced computerized healthcare algorithms, have significantly contributed to the prevalence and widespread adoption of CDSSs [125]. In fact, with the ongoing innovations in artificial intelligence, the CDSS market is expected to grow from $1.3 to $2.2 billion USD in the next 3–5 years [126].

Our findings also revealed that most of the pre-discharge interventions were developed and implemented with three primary goals: to benefit hospitals financially, improve the quality of care provided, and optimize resource utilization. Additional motivators included population health management and regulatory compliance. While these interventions were primarily designed from the hospital’s perspective, they inevitably impact patient care delivery. Notably, only two distinct interventions mentioned patient education as an area needing improvement. This observation highlights a significant gap in prioritizing patient interests in the development and implementation of pre-discharge interventions for early discharge.

While most authors expressed positive sentiment, a significant portion (45%) of the studies reported mixed or negative views on intervention outcomes. This diversity in author sentiment may be attributed to the lack of consistent metrics and inadequate post-development follow-up, which are crucial for evaluating the continuous efficacy and reliability of pre-discharge interventions before widespread adoption. Remarkably, most studies prioritized hospital-centered outcomes, often overlooking patient-centered outcomes—an increasingly important consideration in healthcare evaluation.

As we transition towards a value-based healthcare system focused on whole-person care and patient outcomes, this review underscores the need for further research into how pre-discharge interventions, particularly those utilizing automated decision-making through CDSS, impact the most crucial stakeholder: the patient. Incorporating a patient-centered focus is vital to assess whether early discharge, facilitated by these interventions, poses additional risks to vulnerable, socioeconomically disadvantaged patient groups. These groups already face challenges such as increased mortality and readmission risk, limited access to post-discharge care, and emotional and physical challenges stemming from anxiety about managing their health without adequate support [127, 128].

Balancing hospital and patient interests in the development phase to create consistent guidelines for measuring intervention impact will be essential. Furthermore, advances in patient-centered outcomes research (PCOR) and intervention evaluation can enhance adoption across acute care settings, ensuring that the benefits of early discharge are realized without compromising patient care quality or safety.

### Limitations

We acknowledge that several articles on pre-discharge interventions have been published since 2020, although those we are aware of are not yet in the testing or evaluation phase. Additionally, pre-discharge intervention articles from other database sources may differ in the distribution of early discharge sentiment found in this review. In addition, it is important to emphasize that early discharge sentiment was derived from the positivity or negativity of outcomes described in the articles by their authors; it may be that longer follow-up evaluation of post-discharge patient outcomes could alter the sentiment.

## Conclusions

This review highlights new pre-discharge interventions promoting early discharge of hospitalized older adults continue to be driven by economic and governmental policies. Evaluation of these interventions post-development primarily reflects the hospital perspective. Incorporating the patient perspective in both the development and evaluation of pre-discharge interventions may help hospitals achieve better patient-centered care delivery.

## Supporting information

S1 Checklist Preferred Reporting Items for Systemic reviews and Meta-Analyses extensions for Scoping Reviews (PRISMA-ScR) checklist. (PDF)

S1 Appendix Specific search strategies for PubMed, Embase, and Scopus databases. (DOCX)

S1 Table Demographic characteristics of study populations in included articles. (DOCX)

S2 Table Measured outcomes and quantitative outcomes of included articles. (DOCX)
