# Supplementary material for: Characterizing pre-discharge interventions to reduce length of stay for older adults: A scoping review
Source: PLoS One. 2025 Feb 10;20(2):e0318233. doi: 10.1371/journal.pone.0318233 (PMC11809920; doi:10.1371/journal.pone.0318233)
Supplement: S1 Appendix — (DOCX) [file pone.0318233.s002.docx]

**S1 Appendix**. **Specific search strategies for PubMed, Embase, and Scopus databases.**

**PubMed:** ((((((((((((((((early discharge) AND (reduced length of stay)) OR (reduced LOS)) AND (acute care)) AND (pre discharge intervention)) OR (intervention)) AND (1983-2020)) AND (older adult)) NOT (telephone)) NOT (telemedicine)) NOT (labor)) NOT (mother)) NOT (early follow-up)) NOT (mental health)) NOT (psychiatric)) NOT (delayed discharge)) NOT (follow-up)

**Embase:** ('early discharge' OR 'reduced length of stay' OR 'pre discharge intervention') AND ('acute care' OR 'hospitalization') AND [1983-2020]/py AND ([adult]/lim OR [aged]/lim OR [old aged]/lim)

**Scopus:** (early AND discharge OR reduced AND length AND of AND stay) AND (acute AND care OR hospital) AND older AND adults
