# Supplementary material for: Characterizing pre-discharge interventions to reduce length of stay for older adults: A scoping review
Source: PLoS One. 2025 Feb 10;20(2):e0318233. doi: 10.1371/journal.pone.0318233 (PMC11809920; doi:10.1371/journal.pone.0318233)
Supplement: S1 Table — (DOCX) [file pone.0318233.s003.docx]

**S1 Table.** **Demographic characteristics of study populations in included articles.**

| Authors and Year | Cohort Size | Gender Distribution | Average Age |
| --- | --- | --- | --- |
| Ahmed et al., 2018^98^ | 447 Patients | 46.5% Female, 53.5% Male | 62.3 Years |
| Aicher et al., 2019^115^ | 1697 Patients (631 Control, 1064 Case) | N/A | 67.5 Years |
| Alaraj et al., 2017^123^ | 174 Patients | 67.8 % Female, 32.2% Male | 54.5 Years |
| Allen et al., 2003^45^ | 1166 Patients (622 Pre, 544 Post) | 58.0% Female, 42.0% Male | 72.0 Years |
| Ansari et al., 2018^113^ | 9409 Patients | 50.2% Female, 49.8% Male | 55.2 Years |
| Babb et al., 2017^120^ | 570 Patients (87 Treatment, 481 Control) | 43.5% Female, 56.5% Male | 68.4 Years |
| Bachman et al., 1987^35^ | 1166 Patients (487 Experimental, 779 Control) | N/A | N/A |
| Batlle et al., 2010^68^ | 10005 Patients | 51.3% Female, 48.7% Male | 57.8 Years |
| Beri et al., 2017^79^ | 644 Patients | 15.58% Female, 84.42% Male | 53.7 Years |
| Carr et al., 2014^121^ | 105 Patients | N/A | N/A |
| Carr et al., 2018^60^ | 4341 Patients (1919 Pre, 2422 Post) | N/A | 80.4 Years (Pre), 81 Years (Post) |
| Casale et al., 1998^91^ | 30715 Patients | 42.15% Female, 57.85% Male | 68.4 Years |
| Collier 1995^36^ | 186 Patients | 32.80% Female, 67.20% Male | N/A |
| Crist et al., 1987^104^ | 221 Patients (103 Control, 118 Study) | N/A | 63.7 Years (Control), 64.5 Years (Study) |
| Dalal et al., 2020^111^ | 345 Patients (241 Pre, 104 Post) | 55.07% Female, 44.93% Male | 42.5 Years (Pre), 41.5 (Post) |
| DeLa'O CM et al., 2014^55^ | 740 Patients (385 Pre, 355 Post) | 64.32% Female, 35.68% Male | N/A |
| Eaton et al., 2019^89^ | 136 Patients (37 Pre, 99 Post) | 71.15% Female, 28.85% Male | 34.5 Years (Pre), 35.5 Years (Post) |
| Ebinger et al., 2018^86^ | 967 Patients | 28.54% Female, 71.46% Male | 62.8 Years |
| Eron et al., 2001^92^ | 223 Patients (111 Infectious Disease Patients, 112 Internal Medicine Patients) | 42.16% Female, 57.84% Male | 61.8 Years |
| Farber et al., 2011^52^ | 8094 Patients | 74.7% Female, 25.3% Male | 83.4 Years |
| Fishbane et al., 2007^49^ | 344 Patients (110 Block 1, 119 Block 2, 115 Block 3) | 50% Female, 50% Male | 73.5 Years |
| Flarity et al., 2017^59^ | 845 Patients (571 Pre, 274 Post) | 40% Female, 60% Male | 58 Years |
| Friedman et al., 2008^50^ | 195 Patients | 78.5% Female, 21.5% Male | 85.2 Years |
| Friedman et al., 2013^109^ | 249907 Patients | 40.6% Female, 59.4% Male | 66.3 Years |
| Gayed et al., 2013^54^ | 638 Patients | 5.33% Female, 94.67% Male | 63.5 Years |
| Gheiler et al., 1999^39^ | 1129 Patients | N/A | N/A |
| Gittell et al., 2000^41^ | 338 Providers, 878 Patients | 49% Female, 51% Male | 66.9 Years |
| Gould 2011^96^ | 154 Patients | N/A | N/A |
| Gross 1995^37^ | 47 Patients | N/A | N/A |
| Hamdy et al., 2014^118^ | 193473 Patients | 51.22%-56.55% Female, 48.78%-43.45% Male | 69.5-71.3 Years |
| Hastings et al., 2014^56^ | 127 Patients (92 Enrolled, 35 Other) | 2.37% Female, 97.63% Male | 74.5 Years |
| Hay et al., 1997^64^ | 209 Patients (108 Control, 101 Intervention) | 39.2% Female, 60.8% Male | 64.2 Years |
| Holland et al., 2003^66^ | 991 Patients | 48.7% Female, 51.3% Male | 62.6 Years |
| Holland et al., 2013^71^ | 260 Patients | 54% Female, 46% Male | 54.7 Years |
| Holland et al., 2017^80^ | 222 Patients | 53.2% Female, 46.9% Male | 61.7 Years |
| Horn et al., 1985^103^ | 218 Patients (126 Before, 92 After) | 47.7% Female, 52.3% Male | 68.3 Years |
| Horowitz et al., 2002^122^ | 277 Patients (163 Pre, 114 Post) | 58.5% Female, 41.5% Male | 66.1 Years |
| Hou et al., 2008^65^ | 2571 Patients | 48% Female, 52% Male | 63.1 Years |
| Houck et al., 2004^106^ | 13771 Patients (8388 Within 4 Hours, 5383 After 4 Hours) | 51.8% Female, 48.2% Male | 79.5 Years |
| Ichibori et al., 2019^116^ | 75 Patients (65 VED, 10 ED) | 47% Female, 53% Male | 81.7 Years |
| Jones et al., 2006^47^ | 266 Patients | 45.4% Female, 54.6% Male | 66.2 Years |
| Kaboli et al., 2004^93^ | 1706 Patients | N/A | N/A |
| Kandzari et al., 2003^105^ | 1703 Patients (842 Control, 861 Intervention) | 27.5% Female, 72.5% Male | 58.5 Years |
| Kates et al., 2011^53^ | 193 Patients | N/A | 84.7 Years |
| Keyes et al., 2014^57^ | 7598 Patients (3850 Pre, 3748 Post) | 57% Female, 43% Male | 70.0 Years |
| Kontos et al., 2003^67^ | 1587 Patients (874 Cohort, 713 Control) | 50.6% Female, 49.4% Male | 55.9 Years |
| Kozma et al., 2010^34^ | 1471295 Patients | 53% Female, 47% Male | 69.9 Years |
| Kucenic et al., 2000^42^ | 110 Patients (56 Pre, 54 Post) | N/A | N/A |
| Kupensky et al., 2015^58^ | 202 Patients | 49% Female, 51% Male | 78.7 Years |
| Lee et al., 1999^40^ | 487 Patients (176 Older Than or Equal To 70 Years, 311 Younger Than 70 Years) | 34.3% Female, 65.7% Male | 67 Years |
| Levine et al., 2018^99^ | 300 Patients (169 Pre, 131 Post | 24% Female, 76% Male | 45 Years |
| Mahler et al., 2013^72^ | 1005 Patients | N/A | N/A |
| Mahler et al., 2015^74^ | 1369 Patients | 57.4% Female, 42.6% Male | 53 Years |
| Mahler et al., 2015^75^ | 282 Patients | 52.9% Female, 47.1% Male | 49 Years |
| Mahler et al., 2016^78^ | 282 Patients | 57.4% Female, 42.6% Male | 53.4 Years |
| Mansouri et al., 2011^108^ | 130 Patients (65 Pre, 65 Post) | 3.1% Female, 96.9% Male | 68.2 Years |
| Mayo et al., 1996^38^ | 126 Patients (61 Pre, 65 Post) | 75.5% Female, 24.5% Male | 40 Years |
| Melamed et al., 2020^100^ | 1105 Patients | 51.7% Female, 48.3% Male | 61.8 Years |
| Meneghini et al., 2017^81^ | 980 Patients | 60.6% Female, 39.4% Male | 62.3 Years |
| Mundy et al., 2003^46^ | 458 Patients [227 Intervention, 231 Without Intervention) | 55.9% Female, 44.1% Male | N/A |
| Naughton et al., 1994^90^ | 111 Patients | 56.2% Female, 43.8% Male | 80.1 Years |
| Ni et al., 1999^112^ | 35560 Patients | 53% Female, 47% Male | N/A |
| Nyswonger et al., 1992^117^ | 52 Patients | 59.6% Female, 40.4% Male | N/A |
| Padula et al., 2009^51^ | 50 Patients (25 Cohort, 25 Control) | 54% Female, 46% Male | 80.4 Years |
| Peralta et al., 2020^101^ | 763 Patients (405 Pre, 358 Post) | 37.5% Female, 63.5% Male | 66.9 Years |
| Perry et al., 2020^102^ | 130 Patients (60 Pre, 70 Post) | 25% Female, 75% Male | 45.2 Years |
| Reddy et al., 2001^43^ | 3331 Patients (1707 Cohort, 1624 Control) | 42.5% Female, 57.5% Male | 54.9 Years |
| Reed et al., 2004^94^ | 1069 Patients | 38.7% Female, 61.3% Male | 66 Years |
| Riley et al., 2017^82^ | 282 Patients | 58% Female, 42% Male | 53 Years |
| Rittenhouse et al., 2015^76^ | 752 Patients (415 Case, 337 Control) | 54.8% Female, 45.2% Male | 82.5 Years |
| Rodriguez-Araujo et al., 2018^114^ | 852 Patients (429 Same Day, 423 Over Night) | N/A | 62 Years |
| Rothberg et al., 2010^107^ | 84621 Patients | 61% Female, 39% Male | 69 Years |
| Rozell et al., 2017^83^ | 1814 Patients (802 Training, 1012 Validation) | 59.6% Female, 40.4% Male | 62.3 Years |
| Rudolph et al., 2014^73^ | 1527 Patients | 2% Female, 98% Male | 78.2 Years |
| Shah et al., 2012^70^ | 777 Patients | 51.5% Female, 48.5% Male | N/A |
| Sharkawi et al., 2017^84^ | 228 Patients | 24.6 Female, 75.4% Male | 63.7 Years |
| Shilian et al., 2020^63^ | 1683 Patients | 59.2% Female, 40.8% Male | 71.5 Years |
| Slauenwhite et al., 1998^33^ | 23 Patients | N/A | N/A |
| Snider et al., 2015^119^ | 471319 Patients (103222 Ons, 368097 Non-Ons) | 56.8% Female, 43.2% Male | 76.7 Years |
| Somanchi et al., 2011^69^ | 767 Patients (200 Baseline, 106 Intervention, 461 Control) | 54.3% Female, 45.7% Male | 52.8 Years |
| Soto et al., 2018^61^ | 572 Patients | N/A | N/A |
| Southern et al., 2007^95^ | 9037 Patients (2913 Cohort, 6124 Control) | 58.1% Female, 41.9% Male | 64.9 Years |
| Stopyra et al., 2015^77^ | 282 Patients | 57.4% Female, 42.6% Male | 53.3 Years |
| Stopyra et al., 2017^85^ | 282 Patients | N/A | N/A |
| Tesson et al., 2018^87^ | 966 Patients | 42% Female, 58% Male | 59 Years |
| Treat et al., 2016^110^ | 98 Patients (51 Control, 47 PEG) | 37.8% Female, 62.2% Male | 50.7 Years |
| Walsh et al., 2001^44^ | 194 Patients (67 Pre, 58 Intervention, 69 Control) | N/A | N/A |
| Walsh et al., 2018^88^ | 384 Patients (152 Pre, 232 Post) | 52.1% Female, 47.9% Male | 64.5 Years |
| Weems et al., 2019^62^ | 308 Hospitalists (129 Pre, 179 Post) | N/A | N/A |
| Zemencuk et al., 2006^48^ | 142 Physicians | N/A | N/A |
| Zhu et al., 2016^97^ | 1064 Patients (814 Np Unit Patients, 250 Other Unit Patients) | 52% Female, 48% Male | 59 Years |
