# Supplementary material for: Characterizing pre-discharge interventions to reduce length of stay for older adults: A scoping review
Source: PLoS One. 2025 Feb 10;20(2):e0318233. doi: 10.1371/journal.pone.0318233 (PMC11809920; doi:10.1371/journal.pone.0318233)
Supplement: S2 Table — (DOCX) [file pone.0318233.s004.docx]

**S2 Table.** **Measured outcomes and quantitative outcomes of included articles.**

| Authors and Year | Measured Outcomes | Quantitative Outcome Results |
| --- | --- | --- |
| Ahmed et al., 2018^98^ | (1) Hospital days gained; (2) Progression of care days gained over 12-month period. | (1) Routine weekend procedural services allowed 108 of 447 (24.2%) patients to be discharged earlier than anticipated, resulting in 174 hospital days gained. (2) Procedures were performed earlier than anticipated in 268 of 447 (60.0%) patients resulting in 415 days of progression of care gained over the 12-month period. |
| Aicher et al., 2019^115^ | (1) Average LOS; (2) 30-day readmission rate; (3) Cost of inpatient care (direct variable + direct fixed costs). | (1) The average LOS prior to intervention was 10.8 ± 0.5 days and 8.0 ± 0.2 days post intervention. (2) The number of 30-day readmissions observed prior to intervention were 81 which increased to 106 post intervention. (3) Cost of inpatient care was $32,896 ± $1,413 prior to intervention and statistically did not differ from post intervention which was $31,974 ± $1,020. |
| Alaraj et al., 2017^123^ | (1) LOS; (2) Total hospital cost. | (1) Mean hospital LOS dropped from 21.6 days to 14.1 days. (2) Total hospital costs per SAH patient decreased from $328K to $269K. |
| Allen et al., 2003^45^ | (1) In hospital deaths; (2) Mean LOS; (3) Discharge destination; (4) Readmission rates. | (1) Patients post-SU had a shorter average LOS than pre-SU (4.6 days pre-SU vs 3.8 days post-SU; P<0.0001). (2) Patients post-SU were less often readmitted during the following year (82% pre-SU and 59% post-SU; P<0.0001). (3) Patients post-SU were more often discharged home (50% pre-SU vs 62% post-SU; P<0.0001). (4) The cost-effectiveness of SU care remains to be determined. Analysis of two units has estimated that hospital costs were not increased because of savings from LOS reductions that offset the clinical nurse specialists and medical director costs. |
| Ansari et al., 2018^113^ | (1) Hospital LOS; (2) Readmission rates. | (1) For the overall population, as the yearly average LOS decreased from 7.5 days to 5.8 days, the yearly readmission rate increased from 0.05 to 0.11. |
| Babb et al., 2017^120^ | (1) LOS. | (1) The adjusted odds of high LOS for persons receiving ONS was 2.43 times that for patients not on ONS (P=0.0037), compared to an unadjusted odds ratio of 5.11. |
| Bachman et al., 1987^35^ | (1) Number of activities of daily living (ADL); (2) Frequency of physical and chemical restraints use; (3) Hospital stay complications; (4) LOS; (5) Administratively necessary days and quantity; (7) Readmissions; (8) Nursing home admissions. | (1) Intervention was associated with reduced LOS without decreased quality of care. Analysis of the individual program elements was not completed, so specific aspects causing the study results cannot be identified. |
| Batlle et al., 2010^68^ | (1) LOS by imaging type. | (1) The mean LOS was significantly shorter for those imaged on day -1 or 0 compared with day 1 or 2 for all admissions of greater than or equal to 3 days (8.6 vs 9.0 days, P=0.015). |
| Beri et al., 2017^79^ | (1) Myocardial infarctions (MI) from presentation to 180 days; (2) Death from presentation to 180 days; (3) Cardiac rehospitalizations; (4) Revascularizations. | (1) Copeptin added value to three of the four analyzed endpoints. (2) The 180day MI rate was significantly lower in the group with normal copeptin (0.0% vs 3.0%, p<.001). (3) The 180day death rate was also significantly lower in the normal copeptin group (0.0% vs 1.2%, p=.02). (4) The 180day cardiac rehospitalization rate was significantly lower in the normal copeptin group (3.4% vs 7.1%, p=.04). (5) The revascularization rate at 180days was also lower in the normal copeptin group (4.0% vs 5.3%, p=.47) although the difference was not statistically significant. (6) Low rates of death and MI at 180 days suggest that management of patients may reasonable take place in outpatient setting. |
| Carr et al., 2014^121^ | (1) Avoided radiology services used. | (1) Based on clinician reports of health services avoided using the HIE, it was estimated that the cost savings were of $283,477.69 which were obtained during the 4-month study period. (2) This represents a mean savings of $2699.77 per patient. |
| Carr et al., 2018^60^ | (1) LOS; (2) Complications; (3) Mortality. | (1) After controlling for age, injury severity score, mechanism of injury, and number of complications, mortality was significantly reduced in the POST group 77 years or older (odds ratio, 0.53; 95% confidence interval,0.3–0.87). (2) Hospital LOS was significantly reduced in the POST group age 78 years or older (regression coefficient, −0.55; 95% confidence interval, −1.09 to−0.01). (3) LOS was similar between both groups: 5.9 days for Pre and 5.8 days for Post. |
| Casale et al., 1998^91^ | (1) Mortality; (2) LOS; (3) Revascularization. | (1) Treatment by a cardiologist was associated with approximately a 17% reduction in hospital mortality. (2) Treatment by a cardiologist as compared by a primary care physician was also associated with a significantly lower LOS for both medically treated patients (p<0.01) and those undergoing revascularization (p<0.01). |
| Collier 1995^36^ | (1) Number of neurological complications; (2) ICU requirement; (3) Average operative time; (4) Average LOS; (5) Hospitalization costs. | (1) The average LOS for all patients was 1.27 days. (2) Twenty-nine patients had a LOS greater than 1 day. (3) 18 patients required ICU care, and their average LOS was 3.17 days. (4) One patient died at home on the 28th postoperative day from a myocardial infarction. |
| Crist et al., 1987^104^ | (1) Length of drug therapy; (2) Hospital LOS; (3) Mortality. | (1) The mean duration of therapy was 5.9 and 10.3 days per patient in the study and control group, respectively (p < 0.0001). (2) The mean length of hospital stay was 8.4 days in the study group and 11.8 days for the control (p < 0.005). (3) The hospital costs were $725 less per patient in the study group, which would produce a savings of approximately $640,000 per year at the institution. |
| Dalal et al., 2020^111^ | (1) Intravenous opioid exposures; (2) All routes opioid exposures; (3) Intravenous morphine milligram equivalents/patient day. | (1) Between study periods, a significant reduction in the proportion of patients receiving IVOPIs was observed (43.6% vs 30.8%, p=0.03) as well as for total opioid exposure (15.6 vs 8.5 intravenous morphine mg equivalents/d, p=0.02). (3) Mean LOS was significantly reduced (7.2 vs 5.3 days, p=0.03). (4) During sensitivity analysis, reduction in LOS and readmission rate did not reach statistical significance.(5) It is hypothesized that reduced LOS could be related to decreased time spent weaning IVOPIs if these medications were avoided early in the hospitalization. (6) Additional research is needed to determine long-term benefits of reduced opioid exposure in this population. |
| DeLa'O CM et al., 2014^55^ | (1) LOS. | (1) The mean LOS after the Geriatric Trauma initiation was significantly reduced when admissions to a nontrauma vs dedicated Geriatric Trauma Institute service was evaluated (5.64 vs 4.43 days; p=0.03). (2) The changes in LOS resulted in a charge reduction of 21% (>$775,000.00) in only the first 5 months since the implementation of the Geriatric Trauma Institute. |
| Eaton et al., 2019^89^ | (1) Mean LOS; (2) Number of readmissions; (3) Direct costs per admission. | (1) Use of the assessment reduced mean LOS by 20 days. (2) Total direct costs were reduced by 33%, creating capacity for an additional 333 patients. (3) Number of readmissions increased from 7 to 18. |
| Ebinger et al., 2018^86^ | (1) Post-triage location; (2) LOS; (3) Ejection Fraction; (4) 30-day and 1-year mortalities; (5) post-PCI major adverse cardiovascular events (MACE). | (1) Results confirmed lower rates of complications (low risk 8.3% vs high risk 38.7%; p<0.001) and in-hospital mortality (low risk 0.4% versus high risk 12.5%; p<0.001). (2) Total protocol adherence was 75% and only 62% for low-risk patients. |
| Eron et al., 2001^92^ | (1) Average LOS; (2) 30-day readmissions; (3) Discharge program satisfaction; (3) Time to return to normal ADL. | (1) Patients cared for by the infectious diseases hospitalist had a shorter average LOS (mean difference = 1.7 days), no readmissions, higher satisfaction scores, and a shorter time to return to their activities of daily living. |
| Farber et al., 2011^52^ | (1) LOS; (2) Total Cost; (3) mortality; (4) 7- and 30-day readmission rates. | (1) Mean LOS and total costs were significantly lower for patients in the MACE service compared with the ACE unit service (5.8 vs 7.9 days, p<0.001, and $10,315 vs $13,187, p=0.002). |
| Fishbane et al., 2007^49^ | (1) LOS; (2) Time to oral antibiotics; (3) Time from clinical stability to discharge. | (1) The mean LOS was significantly lower in block 2 (5.3 days) than in blocks 1 (8.8 days) and 3 (7.3 days). (2) The mean time from clinical stability to discharge was significantly shorter in block 2 (2.1 days) than for blocks 1 (5.3 days) and 3 (4.9 days). |
| Flarity et al., 2017^59^ | (1) Hospital LOS; (2) Narcotic usage; (3) Pulmonary function. | (1) Despite being significantly older with more rib fractures in the ICU, patients admitted after implementation of CPG had a significantly reduced LOS by over 2 days. |
| Friedman et al., 2008^50^ | (1) Mean LOS; (2) Readmission rates; (3) Complications rates; (4) Mortality rates. | (1) Mean LOS was 4.6 days, compared with the expected rate of 5.2 days after adjusting for patient characteristics. (2) Mean LOS for individuals from home, assisted living, and nursing homes was not significantly different with stays of 5.0, 4.5 days, and 4.1, respectively (p=0.35). (3) Financial consequences need further evaluations. |
| Friedman et al., 2013^109^ | (1) Demographics; (2) Clinical characteristics; (3) Clinical outcomes; (4) Healthcare resource utilization; (5) Hospitalization costs. | (1) The GRI-only cohort had higher major bleeding rates (3.3%), mortality (7.6%), and costs ($21,975) than the clopidogrel only and triple therapy cohorts. (2) Late-treated clopidogrel-only patients had higher rates of stroke (6.9%), ACS-related re-admissions (6.1%), and all-cause re-admissions (15.9%) than other cohorts. (3) Late treatment was overall associated with longer LOS and significantly higher costs. |
| Gayed et al., 2013^54^ | (1) LOS; (2) non-VA costs. | (1) LOS decreased 36% overall, decreasing from 5.3 days during the pre-project period to 3.4 days during the 20-month sustainment period (p<0.001). (2) Non-VA care was completely eliminated producing an estimated return on investment of $1 million annually. |
| Gheiler et al., 1999^39^ | (1) LOS; (2) Morbidity and mortality; (3) Readmission rate; (4) Costs. | (1) The average length of stay for radical prostatectomy patients decreased significantly from 8.1 days to 4.9 days after implementation of the clinical care pathway. (2) The difference in length of stay between academic and private physicians was eliminated after 1 year of CCP implementation. (3) The average cost of radical prostatectomy decreased by 16% in 1994 and 22% in 1995 after CCP implementation, compared to the 1993 baseline. |
| Gittell et al., 2000^41^ | (1) Quality of care;(2) LOS; (3) Pain and functioning. | (1) Improved quality of care was significantly associated with higher levels of relational coordination among care providers (linear regression coefficient 1.068, p<0.001). (2) LOS were also significantly associated with relational coordination among care providers: a 1-point increase in relational coordination was associated with a 53.77% decrease in LOS. |
| Gould 2011^96^ | (1) Medication adherence; (2) Patient satisfaction; (3) Use of urgent care; (4) Illness perception in patients. | (1) No significant group differences were found on 3 of 4 outcomes. (2) Medication adherence in both groups was extremely high, as was patient satisfaction. (3) Use of urgent care was minimal. (4) Adding additional nursing support, post-procedure, did not influence medication adherence, use of urgent care, or patient satisfaction in the period studied. |
| Gross 1995^37^ | (1) Reintubations; (2) Cardiac arrests; (3) Deaths; (4) Mean LOS; (5) Costs; (6) Resource utilization. | (1) The incidence of reintubation, cardiac arrest, or death was zero. (2) The mean postoperative LOS was 5.8 days. (3) Preliminary findings warrant further research to validate the continued safety and efficacy of practice. |
| Hamdy et al., 2014^118^ | (1) LOS; (2) Resource utilization hospital costs. | (1) Tube-fed patients with DM who were provided GTSN had a 0.88-day shorter LOS on average which contributed to a cost savings of $2,586 for tube-fed patients. (3) However, tube-fed patients receiving GTSN had greater reductions in LOS and costs than orally fed GTSN patients. |
| Hastings et al., 2014^56^ | (1) LOS; (2) Inpatient falls; (3) Discharge destination; (4) ED visits; (5) 30-day hospital readmissions. | (1) Median LOS was 4.7 days for STRIDE participants and 5.7 days for individuals receiving usual care (p=0.31). (2) Thirty-day ED visit and readmission rates were not significantly different between the two groups. |
| Hay et al., 1997^64^ | (1) LOS; (2) Complication rate after achieving low-risk status (in-hospital mortality, 30-day readmissions, and bleeding episodes). | (1) Seventy percent of UGIH patients achieved low-risk status according to the guideline and were therefore potentially suitable for early discharge. (2) Mean LOS decreased from 4.6 days to 2.9 days (mean reduction of 1.7 days per patient; p<0.001). |
| Holland et al., 2003^66^ | (1) Prolonged LOS; (2) Use of nonroutine discharge planning services; (3) Discharge disposition. | (1) Overall PRA score did not effectively screen for endpoints in the study. (2) AUC of PRA ranged from 0.58 to 0.79. |
| Holland et al., 2013^71^ | (1) The Problems After Discharge Questionnaire – English Version (PADQ-E). | (1) Patients with high ESDP scores reported significantly more problems (mean = 16.3) than those with low scores (mean = 12.2). (2) |
| Holland et al., 2017^80^ | (1) The Problems After Discharge Questionnaire; (2) EuroQoL-5Dimensions quality-of-life measure; (3) LOS; (4) Use of post-acute care services. | (1) Patients with high ESDP scores reported more problems after discharge (p=0.02), lower quality of life (p<0.001), had longer LOS (5.0 vs 3.7) (p=0.04), and used post-acute services (p=0.006) more than patients with low ESDP scores. |
| Horn et al., 1985^103^ | (1) Patient LOS; (2) Discharge status; (3) Signs of digitalis toxicity; (4) Time to therapeutic drug level. | (1) The number of digoxin sample obtained was reduced by 22% in the after group and plasma concentrations were less often in the toxic range. (2) The after group had significantly shorter LOS (15.3 vs 11.6 days). (3) Clinical pharmacist intervention was not found to be associated with any significant changes in patient outcomes, including LOS. |
| Horowitz et al., 2002^122^ | (1) Patient knowledge/experience with care; (2) Time on antibiotics lowered; (3) Time to switch from IV. | (1) Mean time on intravenous antibiotics decreased from 5 days to 4.3 days (p=0.04). (2) More patient’s reporter that they received all the information they needed to recover (75% vs 94%, p=0.02). |
| Hou et al., 2008^65^ | (1) LOS; (2) Costs; (3) Need for social work intervention. | (1) Older patients, sicker and less functional, have higher needs for social work intervention (p<0.001). New nursing home placement patients were older and had worse function (p<0.001). (3) Due to tertiary care and the type of patients seen, findings may not apply to other settings. |
| Houck et al., 2004^106^ | (1) Mortality; (2) LOS; (3) 30-day readmission. | (1) Among patients who had not received outpatient antibiotic agents, antibiotic administration within 4 hours of arrival at the hospital was associated with a reduced in-hospital mortality rate (6.8% vs 7.4%). (2) Mean LOS was 0.4 days shorter with antibiotic administration within 4 hours than with later administration. |
| Ichibori et al., 2019^116^ | (1) 30-day all-cause mortality; (2) 30-day all-cause rehospitalization. | (1) Mean LOS was significantly shorter in the VED strategy (3.1 days) than in the ED strategy (4.1 days) (p<0.01). (2) During the 30-day follow-up, a total of 11 deaths and 52 rehospitalizations occurred. |
| Jones et al., 2006^47^ | (1) Ancillary testing; (2) Anticoagulation; (3) Inpatient LOS. | (1) Overall, LOS was significantly shorter for patients admitted through the pathway (43 hours vs 82 hours, p<0.01). (2) Whether LOS reductions were due to pathway or management by cardiologists rather that internists is not known. |
| Kaboli et al., 2004^93^ | (1) Hospital mortality; (2) 30-day readmission rate; (3) Hospital LOS; (4) Hospital costs. | (1) Similar rates of in-hospital mortality (1.3% vs 2.1%, p=0.29) and 30-day readmissions (7.8% vs 8.7%, p=0.55) were observed. (2) mean hospital LOS was 1 day shorter for HP patients in unadjusted analyses (5.5 vs 6.5 days, p=0.009). (3) Mean costs per day were $122 higher for HP patients (p=0.003). |
| Kandzari et al., 2003^105^ | (1) LOS; (2) In-hospital deaths; (3) Reinfarctions; (4) Ischemic target vessel revascularization (TVR); (5) Disabling strokes’ (6) Total in-hospital costs. | (1) Hospitalization was significantly shorter in abciximab-treated patients (3.1 vs 3.5 days, p<0.001), but total in-hospital costs did not differ significantly ($13,413 vs $13000, p=0.13). |
| Kates et al., 2011^53^ | (1) Profit/Loss; (2) LOS; (3) Mortality; (4) Complication rates; (5) Hospital readmission. | (1) Adjusting for patient characteristics, costs are demonstrated to be 66.7% of the expected costs nationally. (2) LOS was noted to be below national averages with a value of 4.6 days. |
| Keyes et al., 2014^57^ | (1) Inpatient Admission; (2) LOS; (3) Emergency department recidivism. | (1) There was no significant difference in time to return within 30 days, 180 days, or average LOS. (2) Risk of being admitted on the index visit was lower for seniors treated in the senior ED compared with the regular ED (relative risk = 0.93). |
| Kontos et al., 2003^67^ | (1) Myocardial infarction prevalence: (2) 30-day revascularization rates; (3) Triaged level; (4) Expenses incurred within 30 days of index visit; (5) ECG characteristics; (6) LOS. | (1) Mean costs per encounter were reduced for the ACT patients for each level, which was significant when all patients were compared ($5,030 vs $6,044, p=0.02). (2) Use of MPI in the low-risk patients was associated with reduced costs (level 3, $4,959 vs $5,051; level 4, $1,529 vs $1,794) and was associated with a significantly lower angiography rate and shorter LOS. |
| Kozma et al., 2010^34^ | (1) Cost per day of CAP; (2) Hospitalization. | (1) Only when medically appropriate, a reduction in the LOS of a CAP hospitalization by 1 day could decrease costs by $2,273-2,373. |
| Kucenic et al., 2000^42^ | (1) Hospital admission; (2) Mortality; (3) LOS; (4) Cost. | (1) The pathway reduced LOS by 2.2 days and hospital charges by $1,008 without compromising care quality and outcomes. |
| Kupensky et al., 2015^58^ | (1) LOS; (2) Occurrence of advanced directive discussion; (3) Occurrence of code status update; (4) Symptom management; (5) Discharge disposition; (6) Abbreviated Injury Scores; (7) Injury Severity Scores. | (1) Total hospital LOS was greatly reduced for patients with a PMC on or before PTD 2 (7.92 vs 13.11, p=0.001). |
| Lee et al., 1999^40^ | (1) Post-operative atrial fibrillation incidence; (2) LOS. | (1) Overall stroke rate 1.4% but the incidence was significantly higher in the older cohort of patients (2.9% vs 0.03, p<0.05). (2) Prevalence rate of postoperative atrial fibrillation among the elderly patients was also twice that observed in the younger group (27% vs 13.7%, p=0.001). |
| Levine et al., 2018^99^ | (1) LOS; (2) Medication use; (3) Surgical interventions; (4) Readmission rates. | (1) The creation of a MTS was associated with a significant reduction in the mean LOS (69.5 hours vs 48.1 hours). (2) However, this reduced LOS was not associated with any statistically significant change in readmission rates. |
| Mahler et al., 2013^72^ | (1) North American Chest Pain Rule (NACPR) score; (2) HEART score; (3) Troponin measures at 0 and 3 hours. | (1) The HEART score identified 20% of participants for early discharge with 99% sensitivity for ACS. (2) The HEART score had a net reclassification improvement of 10% versus unstructured assessment and 19% versus NACPR. |
| Mahler et al., 2015^74^ | (1) Early discharge rate; (2) Sensitivity for major adverse cardiac events. | (1) Compared with usual care, use of the HEART Pathway decreased objective cardiac testing at 30 days by 12.1% and LOS by 12 hours. (2) Early discharges increased by 21.3%. |
| Mahler et al., 2015^75^ | (1) Cardiac testing: (2) LOS; (3) Early discharge; (4) MACE. | (1) MACE occurred in 31 patients (2.7%). (2) ADAPT identified 551 (48.3%) patients for early discharge of which 5 had MACE at 30 days. |
| Mahler et al., 2016^78^ | (1) Adherent vs nonadherent care; (2) Index visit disposition; (3) MACE at 30 days; (4) Discharge rate; (5) Number of admissions. | (1) Nonadherence to the HEART Pathway occurred in 28 patients (20%). (2) Over testing occurred in 19 patients (13.5%). (3) Undertesting occurred in 9 patients (6%) causing unsafe discharges. |
| Mansouri et al., 2011^108^ | (1) Antibiotic Use; (2) LOS; (3) Cost savings. | (1) Number of restricted antibiotics ordered was significantly reduced to 12 out of 114 (10.2%) antibiotics ordered in the post-ARP period (p=0.024). (2) Mean LOS was also reduced from 7.6 days to 5.8 days. (3) The ARP was associated with a savings of $943 per patient treated for CAP. |
| Mayo et al., 1996^38^ | (1) LOS; (2) Readmission; [3) Process measures. | (1) LOS for all medical service patients fell by 0.5 days (4%), from 12.3 days to 11.8 days. |
| Melamed et al., 2020^100^ | (1) Inpatient mortality; (2) 30-day mortality; (3) One-year mortality; (4) ICU LOS; (5) Hospital LOS. | (1) Hospital LOS decreased from 4.78 to 2.96 and 2.81 days (p<0.001). (2) Most of the hemorrhagic complications were minor. |
| Meneghini et al., 2017^81^ | (1) Discharge disposition; (2) LOS; (3) All cause 90-day readmissions. | (1) The positive predictive value of the OARA score was 81.6% for the same or next day discharge. |
| Mundy et al., 2003^46^ | (1) LOS; (2) Adverse events; (3) Other secondary outcomes. | (1) Hospital LOS for EM vs usual care was significantly less (5.8 days vs 6.9 days; adjusted absolute difference = 1.1 days). |
| Naughton et al., 1994^90^ | (1) LOS; (2) Total cost; (3) Lab costs; (4) Pharmacy costs; (5) Rehab service costs. | (1) Patients in the intervention group had 2.1 fewer days of hospitalization, but this shorter LOS was not statistically significant (p=0.108). (2) There were no differences in mortality or discharge disposition. (3) In risk-adjusted multiple regression analysis, the intervention group was associated with a lower predicted total cost per patient than the usual care group (-$2,544). |
| Ni et al., 1999^112^ | (1) Hospital admission rate; (2) Length of hospital stay; (3) In-hospital mortality; (4) Hospital readmission for congestive heart failure (CHF) within the same year of discharge. | (1) The average LOS decreased from 5.01 days in 1991 to 3.95 days in 1995. |
| Nyswonger et al., 1992^117^ | (1) LOS. | (1) In patients fed within 72 hours of admission, LOS was 20.14 days. (2) The LOS for patients fed later than 72 hours from the time of admission was 29.76 days. (3) The difference was statistically significant (p=0.036) but further study is warranted to ensure that early enteral nutrition is the factor driving decreases in LOS. |
| Padula et al., 2009^51^ | (1) Mobility - Barthel Index; (2) ' UP and Go Test'; (3) LOS. | (1) Treatment group had significantly shorter LOS (4.96 days vs 8.72 days; p<0.001). |
| Peralta et al., 2020^101^ | (1) LOS; (2) Unplanned readmission rates; (3) Hospital financial metrics. | (1) Implementation of CDR resulted in a 23% decrease in LOS (p=0.003). (2) 30-day unplanned readmission rates did not change in the 12 months after CDR was initiated compared with the year before (p=0.92). |
| Perry et al., 2020^102^ | (1) LOS; (2) Time to tracheostomy; (3) Days to evaluation; (4) 30-day readmission; (5) 30-day mortality. | (1) Median LOS decreased by 1 week from 26 days to 19 days (27%, p<0.001) with the implementation of the mPATH team. (2) The median time to evaluation by occupational, physical, and speech therapy was universally decreased, by6 60%, 40%, and 33%, respectively. |
| Reddy et al., 2001^43^ | (1) Hospital costs; (2) LOS; (3) resource use. | (1) For patients with CAP, significant decreases in average adjusted cost were observed ($8,164 vs $6,282, p=0.15). (2) LOS of CAP patients decreased from 5.0 days to 4.2 days, p=0.04). (3) However, these effects were identical for the hospitalist and nonhospitalists groups. |
| Reed et al., 2004^94^ | (1) LOS; (2) Readmission; (3) Mortality; (4) Endovascular treatment. | (1) LOS in 2000 was 8.5 days for all diagnostic categories. (2) In 2001, LOS averaged 5.9 days per patient, a reduction in LOS of 2.6 days per patient (p<0.001). (3) In 2002 overall LOS averaged 5.6 days per patient, a reduction of 2.9 days (33%) compared to the standard group. |
| Riley et al., 2017^82^ | (1) Facility costs; (2) Professional costs. | (1) The HEART Pathway arm had a statistically significant lower cost (median cost $1,307) per individual compared with the usual care arm (median cost $1,523). |
| Rittenhouse et al., 2015^76^ | (1) Patient stability on discharge; (2) Discharge to hospice rates; (3) Mortality; (4) Time to care processes; (5) Total time in emergency department; (6) LOS. | (1) ACT patients had significantly shorter median times from ED arrival to INR (13 minutes vs 80 minutes, p<0.001) and to head CT (35 minutes vs 65 minutes, p>0.001). (2) Of the admitted patients, ACT had a significantly shorter median LOS (3.7 days vs 5.0 days, p<0.001). |
| Rodriguez-Araujo et al., 2018^114^ | (1) 30 day-MACE; (2) Procedure-related complications; (3) Total operative costs. | (1) Procedural costs differences of $3,346.45 versus $4,681.99 (p<0.0001) were observed for same day discharge versus and overnight and $4,493.22 versus $7,112.21 (p<0.0001) for 30-day post-procedure-associated cumulative costs. |
| Rothberg et al., 2010^107^ | (1) Risk of treatment failure; (2) LOS; (3) Hospitalization costs. | (1) Treated patients were less likely than nontreated patients to receive mechanical ventilation after the second hospital day (1.07% vs 1.80%), had lower rates of mortality (1.04$ vs 1.59%), and lower rates of readmissions for acute exacerbations of COPD (7.91% vs 8.79%). (2) Patients treated with antibiotic agents had a higher rate of readmissions for Clostridium difficile (0.19%) than those who were not treated (0.09%). |
| Rozell et al., 2017^83^ | (1) Late complications associated to total knee or total hip replacement surgeries. | (1) 382 patients experienced a complication postoperatively. (2) of these, 152 required active management. (3) Multiple logistic regression analysis identified cirrhosis (OR=5.89, p=0.044), congestive heart failure (OR=3.12, p=0.002), and chronic kidney disease (OR=3.85, p<0.001) as risk factors for late complications. |
| Rudolph et al., 2014^73^ | (1) LOS; (2) Restraint use; (3) Discharge to rehabilitation; (4) Hospital variable direct costs. | (1) Patients with interventions were discharged to rehabilitation similarly (mean difference = 2.2%) and had lower length of stay (mean difference = -0.7 days), lower restraint use (mean difference = -4.0%) and trended toward lower variable direct costs (mean difference = -$1,390). |
| Shah et al., 2012^70^ | (1) CPOU usage costs; (2) LOS; (3) Hospital cost savings. | (1) The expected LOS based on this model was 418.5 days versus the actual LOS of 1324 days. (2) Annual cost savings were estimated to be $1,236,832. |
| Sharkawi et al., 2017^84^ | (1) Adverse clinical events; (2) Mortality; (3) LOS. | (1) Low risk patients had lower major adverse clinical events at day 3 or later (0 vs 11.4%, p=0.0002) and had lower total mortality at 1 year (0 vs 4.8%, p=0.02) than patients with intermediate to high risk. |
| Shilian et al., 2020^63^ | (1) Average LOS. | (1) Mean LOS with daily ICCs was 3.37 days compared with 5.55 days in the hospital without daily ICCS (p>0.0001). |
| Slauenwhite et al., 1998^33^ | (1) Cognitive status; (2) Functional independence measure; (3) Pain; (4) Quality of life; (5) Patient satisfaction. | (1) Reduced LOS was not a major issue for 15 of the 23 families studied. |
| Snider et al., 2015^119^ | (1) LOS; (2) Hospital costs; (3) 30-day readmissions. | (1) In unadjusted comparisons in the matched sample, ONS use was associated with longer LOS (8.7 days vs 6.9 days, p<0.001) and lower readmission rates (24.8% vs 26.6%, p=0.0116). |
| Somanchi et al., 2011^69^ | (1) LOS; (2) Percentage/severity of malnutrition; (3) CMI; (4) DRG coding; (5) Time to nutrition support implementation. | (1) Multiple general linear regressions showed that nutrition intervention reduced LOS an average of 1.93 days in the cohort group and 3.2 days in the severe malnourished group. |
| Soto et al., 2018^61^ | 1) ED discharge rate; (2) 30-day ED readmission rate; (3) 30-day ED revisit rate; (4) Hospital management costs. | (1) Among patients presenting with cardiac-related complaints, rates of discharge from the ED increased from 44.4 to 56.6% (p < 0.0001). (2) The net institutional financial impact-accounting for program costs and the explicit revenue gain/losses-was a loss of $171,370 in the 12-months following HEART TRACKS implementation, or approximately $300 per patient. |
| Southern et al., 2007^95^ | (1) LOS; (2) 30-day readmission rate; (3) In-hospital mortality rate; (4) 30-day mortality rate. | (1) Mean LOS was lower for teaching hospitalists than for non-hospitalists (5.01 vs 5.87 days, p<0.02). (2) This LOS reduction was greatest for patients requiring close monitoring (patients with congestive heart failure, stroke, asthma, or pneumonia) and for those requiring complex discharge planning. (3) No significant differences between groups in readmission, in-hospital mortality, or 30-day mortality. |
| Stopyra et al., 2015^77^ | (1) MACE within 30 days. | (1) The EDACS-ADP identified 188/282 patients (66.7%, 95% CI 60.8-72.1%) as low-risk. Of these, 2/188 (1.1%, 95% CI 0.1-3.9%) had MACE at 30 days. (2) EDACS-ADP was 88.2% (95% CI 63.6-98.5%) sensitive for MACE, identifying 15/17 patients with MACE. (3) Specificity was 70.2% (95% CI 64.3-75.6%), PPV (positive predictive value) was 16.0% (95% CI 9.2-25.0%), and NPV was 98.9% (95% CI 96.2-99.9%). |
| Stopyra et al., 2017^85^ | (1) MACE. | (1) In the HEART Pathway arm, both NOTR and HEART Pathway identified all patients with MACE as at risk. (2) Compared to NOTR, the HEART Pathway was able to correctly reclassify 27 patients without MACE as low risk, yielding a NRI of 20.8%. |
| Tesson et al., 2018^87^ | (1) 42-day MACE. | (1) HEART Pathway defined 384 patients as low risk (39.8%) and eligible for early discharge. (2) Applying HEART Pathway would have missed 1.2% of patients with MACE. (3) However, all adverse cardiac events occurred in patients with a HEART Pathway score of 3 (4 of 193, 2.1%) and none in those with a HEART Pathway score ≤2 (0 of 134). (4) Patients with a score of 3 may have higher risk of 42-day MACE that may be unacceptable to some providers, while scores ≤2 saw no events. Caution is advised for those with HEART Pathway score of 3 until more data is available to accurately estimate risk. |
| Treat et al., 2016^110^ | (1) Time to bowel movement; (2) LOS; (3) Readmissions rates; (4) Patient reported pain; (5) Total opioid analgesic use; (6) Postoperative complications. | (1) The primary outcomes measure, time to bowel movement, was significantly shorter than the control group by an entire day (2.9 days vs 4.0 days; p<0.001). (2) In propensity score analysis, patients receiving PEG had bowel movements sooner (-1.06 days; p<0.001) and decreased lengths of stay (-1.16 days; p<0.001). |
| Walsh et al., 2001^44^ | (1) LOS. | (1) Use of nurse care manager significantly reduced physician-related deviations, from the pathway from 10% to 0% (p=0.015). (2) This also reduced system-related deviations from 3% to 0%. (3) Median postoperative LOS was 7 days before the pathway begun, 6 days with the original pathway, and 5 days after the introduction of a vascular nurse specialist (p=0.001). |
| Walsh et al., 2018^88^ | (1) Duration of therapy; (2) LOS; (3) 30-day readmission rates related to pneumonia. | (1) When compared with the preintervention group, mean duration of therapy decreased (9.9 vs 6.0 days; P < .001). (2) More patients received an appropriate duration of 7 days or less (26.9% vs 66.4%; P < .001). (3) Additionally, mean hospital length of stay decreased in the postintervention group (4.9 vs 3.5 days; P= .006). |
| Weems et al., 2019^62^ | (1) Clinical practice; (2) Patient outcomes; (3) Costs. | (1) Intrafacility care variation seen among Novant Health's 11 facilities employing hospitalists decreased from 14.9% to 8.5%, and overall quality-of-care scores by individual providers improved by 14.6 percentage points from study start to end. (2) Care standardization around evidence-based practices, as measured by the simulations, was associated with appreciable decreases in patient length of stay and readmissions, amounting to nearly $1.1 million in savings for Novant Health. |
| Zemencuk et al., 2006^48^ | (1) LOS; (2) Physician satisfaction. | (1) LOS at the profiled site decreased by an additional 1/3 of a day in the profiling year, compared to the non-profiled sites (p<0.001). |
| Zhu et al., 2016^97^ | (1) Diagnostic test utilization; (2) Average LOS; (3) 90-day readmission rate. | (1) The average length of stay was shorter for patients in the NP unit (2.7±3.6 days compared to 3.9±3.4 days, p<.0001). (2) Additionally, the 90-day readmission rate was less for patients in the NP unit (2.7% vs. 3.9%, p<.0006). (3) Statistical design of study may need to be revised. |
